# A dedicate sensorimotor circuit enables fine texture discrimination by active touch

**DOI:** 10.1371/journal.pgen.1010562

**Published:** 2023-01-17

**Authors:** Jie Yu, Xuan Guo, Shen Zheng, Wei Zhang

**Affiliations:** 1 School of Life Sciences, IDG/McGovern Institute for Brain Research, Tsinghua University, Beijing, China; 2 Tsinghua-Peking Center for Life Sciences, Beijing, China; University of California, Santa Barbara, UNITED STATES

## Abstract

Active touch facilitates environments exploration by voluntary, self-generated movements. However, the neural mechanisms underlying sensorimotor control for active touch are poorly understood. During foraging and feeding, *Drosophila* gather information on the properties of food (texture, hardness, taste) by constant probing with their proboscis. Here we identify a group of neurons (sd-L neurons) on the fly labellum that are mechanosensitive to labellum displacement and synapse onto the sugar-sensing neurons via axo-axonal synapses to induce preference to harder food. These neurons also feed onto the motor circuits that control proboscis extension and labellum spreading to provide on-line sensory feedback critical for controlling the probing processes, thus facilitating ingestion of less liquified food. Intriguingly, this preference was eliminated in mated female flies, reflecting an elevated need for softer food. Our results propose a sensorimotor circuit composed of mechanosensory, gustatory and motor neurons that enables the flies to select ripe yet not over-rotten food by active touch.

## Author summary

The physical property is essential for us to access the palatability of food. We tend to feed on food within certain range of hardness. The difficulty to masticate or swallow indicates that the food is unripe or not well-cooked. On the contrary, food that are too soft or viscous could be a sign of over-ripen or contamination of pathogenic microbes. The texture of food is sensed by the mechanosensors on the lips and tongues but the molecules and neurons mediate the sensation are largely unknown. In the current study, we report that fruit flies are most attracted to chewy food, rather than those with too low or too high stiffness. A group of mechanosensory neurons on the fly proboscis are activated during active probing on food surface and promote ingestion by activating the sweet-sensing neurons. These neurons also activate motor neurons to facilitate food ingestion. The texture preference is versatile as mated female flies switch their preference toward softer food.

## Introduction

Animals generate voluntary movements of their sensory organs to explore the environment. For example, during food searching and chewing, specialized mechanosensory receptors on the hands and tongues gather information about the properties of food (texture, hardness, chewiness, etc.). Proprioceptive signals encoding joint movements and positions, arise from muscle engaged in chewing are also involved in this process. Simultaneous cutaneous and proprioceptive information from the food elicits haptic feedback to the brain in order to evaluate the physical property of the food.

The physical property is essential for animals to access the palatability of a food source [1–4]. Animals tend to feed on food within certain range of hardness. The difficulty to masticate or swallow indicates that the food is unripe or not well-cooked. On the contrary, food that are too soft or viscous could be a sign of over-ripen or contamination of pathogenic microbes [5–8]. Thus, together with chemical signals, the textural properties of food provide vital information of the ingestibility and digestibility before the food is ingested.

In human, the physical properties of food are assessed by sensory neurons innervating the tongue, mouth cavity and pharynx [9]. However, the molecular and cellular mechanisms underlying this sensation are largely elusive. A line of studies using *Drosophila* have provided valuable insights into how the mechanical information during food engagement and ingestion is sensed and processed [10–12] and how it is integrated into the feeding control circuit to coordinate food intake [10,13–15]. The mechanosensory neurons underneath the taste sensilla are activated when the labellum contacts food substrate above certain stiffness range and this activation suppresses feeding by inhibiting the sugar-sensing gustatory neurons [10]. The labellum multi-dendritic neurons (md-L) that innervate majority of the sensilla employ *dTmc* to sense the hardness or viscosity of food so as to suppress feeding [11]. However, in most of these studies, flies were allowed to choose between soft (0.25~0.5%, measured as agarose concentration) and hard (1~2%) food sources. While the soft end felled into the range of the food patches that flies were most likely to feed in the natural environment, the hard end was usually beyond the limit that was seen for optimal food sources [10–12]. Despite earlier attempts to establish the rough range of food hardness that the flies prefer, the most palatable stiffness range of food remains unclear and awaits further characterization.

Besides the chemical and mechanical cues from food, female flies’ feeding behavior is also regulated by reproductive states. It was reported that a newly mated female fly was more attracted to food rich in yeast and polyamines [16–19]. Mated flies also exhibit a higher preference to acid and salt [20,21]. This post-mating switch of feeding preference is essential for the egg development [22–25]. However, whether the preference for food stiffness is also subjected to post-mating regulation is unknown.

In the current study, we report that fruit flies are most attracted to chewy food, rather than those with too low or too high stiffness. A group of mechanosensory neurons on the proboscis are activated during active probing by moderate stiffness and promote ingestion by activating the sugar-sensing neurons. These neurons also activate motor neurons to facilitate food ingestion. This preference is regulated by mating states and may aid flies the ability to avoid food that are too soft or watery so that they are away from overripe food or the risk of being stuck.

## Results

### *Drosophila* discriminates fine texture difference during feeding

**Fig 1:**
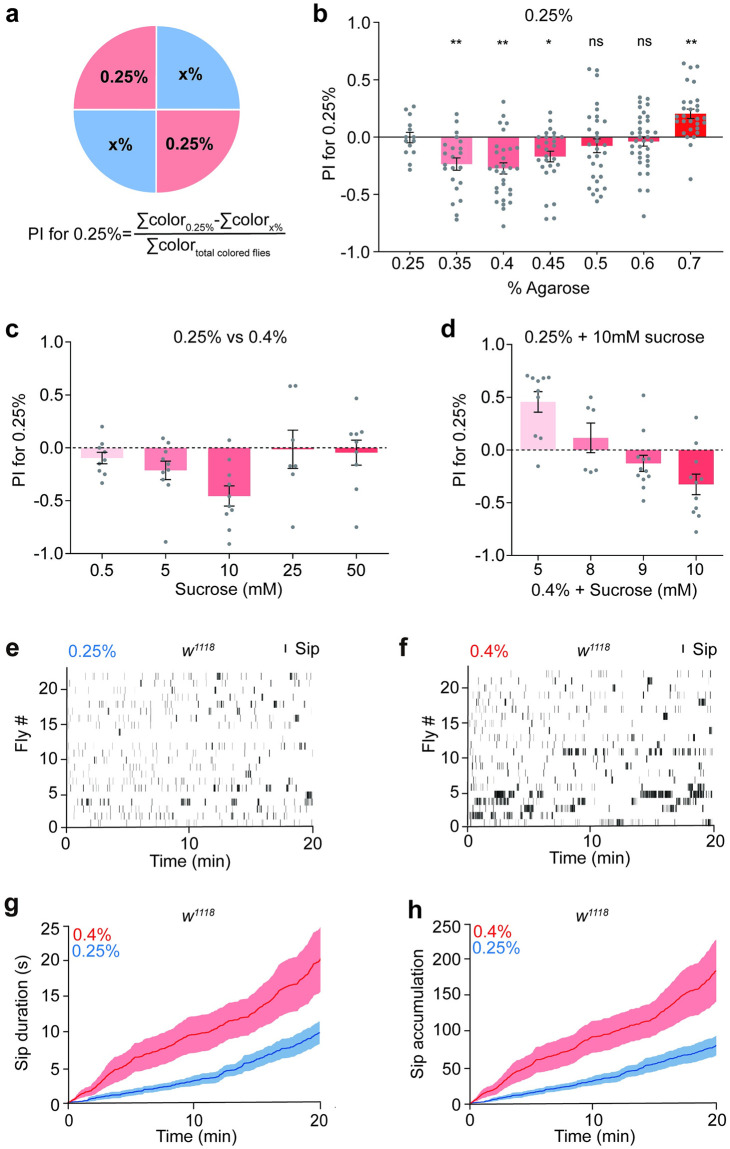
Discrimination of fine texture difference during feeding. **a** Experimental diagram for fine texture preference of feeding. A two-way choice behavioral assay was performed in an arena with two different concentrations of agarose containing red/blue food dyes. A preference index (PI) for 0.25% agarose was calculated based on the color of the fly abdomen for each stiffness. **b** The preference of food hardness of *w*^*1118*^ males in the two-way choice feeding assay. PI for 0.25% of *w*^*1118*^ tested under 7 ranges of stiffness difference (0.25% vs 0.25%~0.7%). 10 mM sucrose was added to different concentrations of agarose. Each gray point represents one independent trial and the number of points per bar indicates the number of replications in each experiment. n = 14, 22, 30, 27, 30, 35 and 29 for each group; Statistical test: one-way ANOVA with Dunnett’s correction for multiple comparisons against the 0.25% group; mean ± SEM. Statistical differences are represented as follows: ns, not significant, p > 0.05; *, p < 0.05; **, p < 0.01. **c, d** Dose-dependent effects of sucrose on stiffness choice (0.25% vs. 0.4%) of *w*^*1118*^ males. (**c**) In each group, flies selected different stiffness between 0.25% and 0.4% with the same sucrose concentration, 5 concentrations (0.5 ~ 50 mM) of sucrose concentration were tested. n = 10, 8, 10, 8 and 8 for each group; (**d)** In each group, flies selected between 0.25% and 0.4% with different sucrose concentration, n = 10, 6, 12 and 11 for each group; mean ± SEM. **e-h** FlyPAD assay of *w*^*1118*^ male flies. Each vertical bar represents a single sip (**e-f**). Flies were fed with 10 mM sucrose mixed with 0.25% agarose (**e**) or 0.4% agarose (**f**); n = 22 for each group. Cumulative sip durations on 0.25% or 0.4% agarose (**g**). Cumulative sip numbers on 0.25% or 0.4% agarose (**h**).

To determine whether food texture influences *Drosophila* feeding choice, we first adapted a two-way choice assay [12] in which flies were allowed to feed on a four-quadrant circular arena which contained sugar food (10 mM sucrose unless otherwise noted was added) dissolved in different concentrations of agarose (Fig 1a). Considering that fruit flies prefer to feed on fully ripe fruits which are normally softer than 0.5% agarose [12], we chose agarose concentrations ranging from 0.25% to 0.7% to best reflect natural food. And the lower end was set to 0.25% agarose, as it became watery and sticky below that concentration. Distinct food dyes were added to adjacent quadrants to facilitate the assessment of ingested amounts of flies by marking the color of their abdomens (all the detailed information of food dyes, sugar and agarose were listed in Table 1). The colors of the various agarose concentrations were random in order to remove side bias. We then calculated a preference index (PI) for the 0.25% agarose (Fig 1a). Unexpectedly, this experiment revealed an obvious preference for feeding on harder food (0.4%) to the softer agarose (0.25%) (Fig 1b). The propensity to choose harder food reached the maximum at 0.4% agarose and then decreased gradually, suggesting that 0.4% agarose is an optimal hardness for flies. When the concentration difference was 0.25% versus 0.7%, flies showed a preference for softer food, consistent with earlier works [11,12]. To exclude the potential influence of food dyes in the two-way choice assay, we switched dyes and found that flies preferred the harder side no matter which color was added (S1a Fig).

**Table 1:** A list of reagent or resource used in this work.

REAGENT or RESOURCE	SOURCE	IDENTIFIER
**Antibodies**		
Goat anti-Rabbit ALexA 488	Invitrogen	Cat#A11008; RRID:AB_143165
Goat anti-Rabbit ALexA 555	Invitrogen	Cat#A-21428; RRID:AB_2535849
Goat anti-mouse ALexA 647	Invitrogen	Cat#A-21235; RRID:AB_2535804
Mouse monoclonal anti-GFP	Sigma	Cat#G6539-100ul; RRID:AB_259941
Rabbit polyclonal anti-GFP	Invitrogen	Cat#A-11122; RRID:AB_221569
Rabbit polyclonal anti-RFP	Rockland	Cat# 600-401-379S; RRID:AB_11182807
Mouse monoclonal anti-nc82	Developmental Studies Hybridoma Bank	RRID: AB_2314866
**Chemicals, Peptides, and Recombinant Proteins**		
Food blue NO.1	TCI	F0147; CAS: 3844-45-9
Food red NO.106	TCI	F0143; CAS: 3520-42-1
Agarose RA	Amresco	Cat#N605-100G; CAS: 9012-36-6
sucrose	Amresco	0335-1KG; CSA: 57-50-1
adenosine 50-triphosphate (ATP)	Sigma	A9187-1G; CSA: 74804-12-9
all *trans*-Retinal	Sigma	R2500-500MG; CSA: 116-31-4
4% PFA	Dingguo Biotech	Cat#AR-0211; CAS: 30525-89-4
Propionic acid	Sigma-Aldrich	402907-500ML; CAS: 79094
**Experimental Models: Organisms/Strains**		
*w* ^*1118*^	Bloomington Drosophila Stock Center	RRID:BDSC 5905
iav-Gal4	Bloomington Drosophila Stock Center	RRID:BDSC 52273
iav-LexA::p65	Bloomington Drosophila Stock Center	RRID:BDSC 52246
*iav* ^*1*^	Laboratory of Yuh-Nung Jan, UCSF	N/A
iav-DBD	Laboratory of Wei Zhang, THU	N/A
vGluT-AD	Laboratory of Yi Rao, PKU	N/A
vGluT-QF, QUAS-mCD8-GFP	Bloomington Drosophila Stock Center	RRID:BDSC 60315
vGluT-gal80	Laboratory of Yi Zhong, THU	N/A
UAS-iav	Laboratory of Wei Zhang, THU	N/A
*nan* ^*Gal4*^	Bloomington Drosophila Stock Center	RRID:BDSC 68205
*tmc* ^*1*^	Bloomington Drosophila Stock Center	RRID:BDSC 66556
*tmc-s* ^*Gal4*^	Laboratory of Yanmeng Guo, UCSF	N/A
tmc-GAL4	Bloomington Drosophila Stock Center	RRID:BDSC 66557
*Gr5a* ^*LexA*^	Laboratory of Hubert Amrein, TAMU	N/A
Gr5a-Gal4	Bloomington Drosophila Stock Center	RRID:BDSC 57592
R66B05-Gal4	Bloomington Drosophila Stock Center	RRID:BDSC 39389
R66B05-LexA	Bloomington Drosophila Stock Center	RRID:BDSC 54917
*Gr33a* ^*Gal4*^	Bloomington Drosophila Stock Center	RRID:BDSC 31425
LexAop-GCaMP6m	Bloomington Drosophila Stock Center	RRID:BDSC 44590
UAS-P2X_2_	Laboratory of Yufeng Pan, SEU	N/A
20xUAS>dsFRT>chrimson-mVenus	Laboratory of Wei Zhang, THU	N/A
8×LexAop2-FLPL	Bloomington Drosophila Stock Center	RRID:BDSC 55820
UAS-CD4-tdTomato (III)	Bloomington Drosophila Stock Center	RRID:BDSC 35837
UAS-CD4-tdTomato (II)	Bloomington Drosophila Stock Center	RRID:BDSC 35841
10×UAS-IVS-mCD8::GFP (II)	Bloomington Drosophila Stock Center	RRID:BDSC 32186
10×UAS-IVS-mCD8::GFP (III)	Bloomington Drosophila Stock Center	RRID:BDSC 32185
UAS-*trans*-tango	Bloomington Drosophila Stock Center	RRID:BDSC 77123?
UAS-CD4-spGFP1-10; LexAop-CD4-spGFP11	Bloomington Drosophila Stock Center	RRID:BDSC 58755
UAS-CsChrimson	Bloomington Drosophila Stock Center	RRID:BDSC 55136
UAS-mCherry.NLS	Bloomington Drosophila Stock Center	RRID:BDSC 38424
R18B07-LexA	Bloomington Drosophila Stock Center	RRID:BDSC 52526
R45G01-LexA	Bloomington Drosophila Stock Center	RRID:BDSC 54866
R81B12-LexA	Bloomington Drosophila Stock Center	RRID:BDSC 54389
VT050405-p65.AD; VT007068-GAL4.DBD	Bloomington Drosophila Stock Center	RRID:BDSC 66875
VT3280-Gal4	Laboratory of Barry J. Dickson, Howard Hughes Medical Institute	N/A
**Software and Algorithms**		
Fiji	NIH Image	https://imagej.nih.gov/ij/
GraphPad Prim 7	GraphPad Software	https://www.graphpad.com/
Anaconda+	Anaconda Inc.	https://www.anaconda.com/
Illustrator	Adobe	https://www.adobe.com/cn

In order to optimize the experimental paradigm, we tried different sucrose concentrations and found that 10 mM sucrose yielded the most significant results in our two-way choice feeding assay (Fig 1c). Although 5 mM sucrose is more consistent than 10 mM according to the SEM value (Fig 1c), the preference for harder food (0.35% ~ 0.45% agarose) was more significant when added 10mM sucrose (S1b Fig versus Fig 1b). When agarose was less sweet, flies ate too little to distinguish the color of their abdomens; when it was too sweet, sucrose overrode their dislike for softer food. Since sweet affects food hardness preference [10,12], we asked whether decreased sweetness and hardness competes with each other. When given 0.25% versus 0.4% agarose with identical 10 mM sucrose, flies preferred the harder side. As the sucrose level in food decreased, flies preferred sweeter food regardless its softness (Fig 1d). These observations suggest that flies are able to discriminate a subtle difference as small as 0.1% (e.g., 0.25% versus 0.35%), demonstrating that the food texture-guided feeding site selection is a deliberate decision. Moreover, we found that flies preferred food within a certain stiffness range and the interaction between food sweetness and hardness is reciprocal.

We then validated these findings by using an automatic feeding monitoring system (FlyPAD) [26] to quantify the total duration of the fly sip within 20 minutes. Wild type *w*^*1118*^ flies took more sips on 0.4% agarose than 0.25% (Fig 1e–1h), consistent with previous results. We concluded that fruit flies can discriminate fine texture difference during feeding and prefer 0.4% agarose to 0.25% agarose.

### The TRP channel *iav* is essential for fine texture sensing

**Fig 2:**
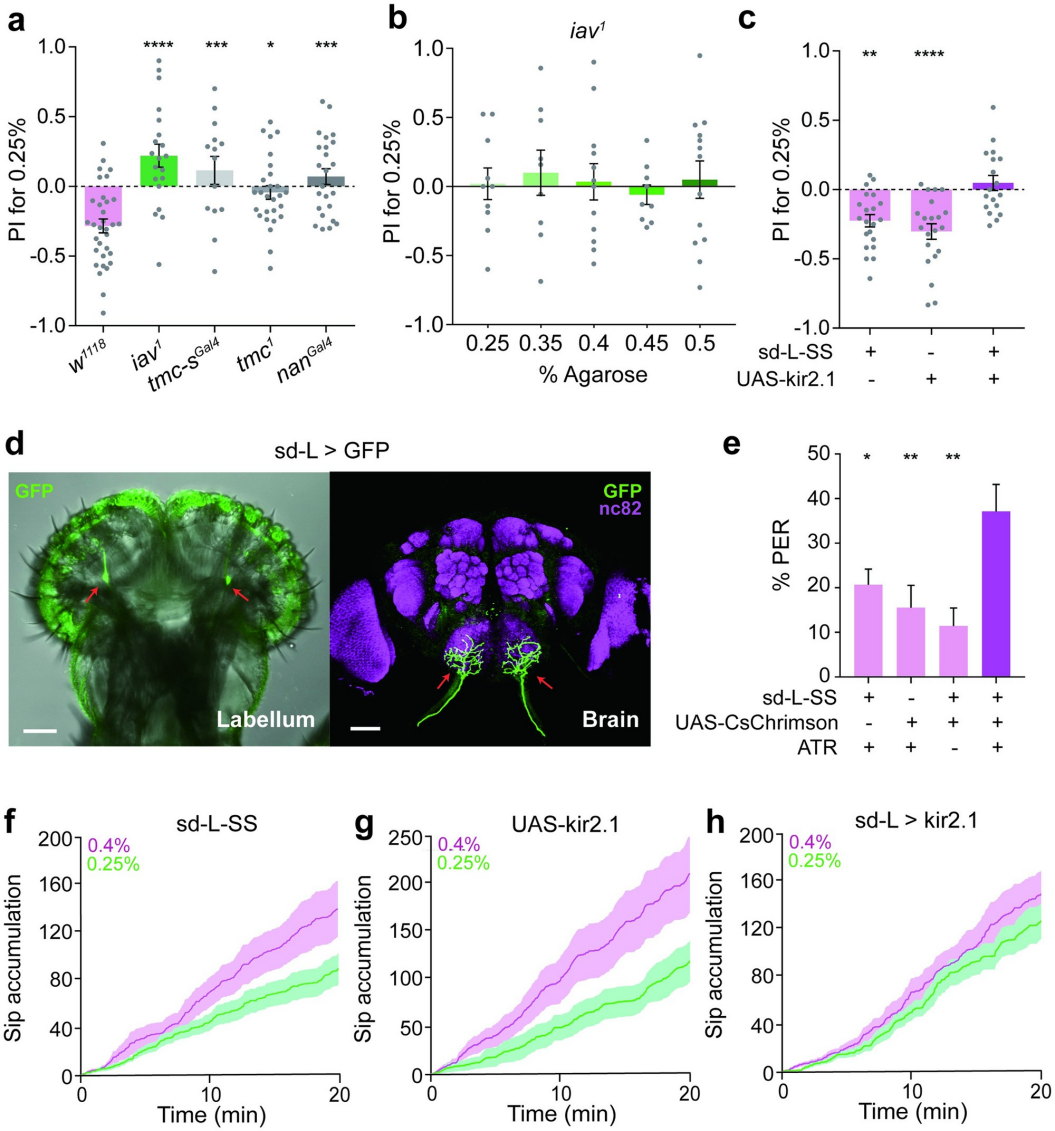
Single-dendritic labellum neurons discriminate substrates of different stiffness during feeding. **a** The preference of food hardness between 0.25% and 0.4% agarose of the mechanotransduction channel gene mutants (*iav*^*1*^, *tmc-s*^*gal4*^, *tmc*^*1*^, *nan*^*gal4*^) in the two-way choice feeding assay. 10 mM sucrose was added to different concentrations of agarose. n = 33, 20, 27, 14 and 25 for each group. Statistical test: one-way ANOVA with Dunnett’s correction for multiple comparisons against the *w*^*1118*^ group; mean ± SEM. **b** The preference of food hardness of *iav*^*1*^ males between 0.25% and 5 different stiffness (0.25%, 0.35%, 0.4%, 0.45%, 0.5%) in the two-way choice feeding assay. 10 mM sucrose was added to different concentrations of agarose. n = 10, 9, 12, 9 and 13 for each group. Data are represented as mean ± SEM. **c** The preference of food hardness when sd-L neurons were silenced with kir2.1 in the two-way choice feeding assay. 10 mM sucrose was added to different concentrations of agarose. n = 21, 21 and 18 for each group. Statistical test: one-way ANOVA with Dunnett’s correction for multiple comparisons against the sd-L-SS > UAS-kir2.1 group; mean ± SEM. **d** Expression patterns for sd-L-SS (split-Gal4: vGluT-AD and iav-DBD) in the labellum and brain. Immunostaining used either anti-GFP and/or anti-Brp (magenta). Scale bar, 50 μm. Brain was counter-stained with the neuropil marker nc82 (magenta). Red arrow pointed to sd-L neurons (in the labellum) and its axon (in the brain). **e** PER assay of flies when sd-L neurons were activated using CsChrimson by exposure to 1 mW/cm^2^ light (595nm). Flies were tested with 20 mM sucrose. n = 29, 18, 14 and 21 for each group. Statistical test: one-way ANOVA with Dunnett’s correction for multiple comparisons against the sd-L-SS > UAS-CsChrimson with ATR group; mean ± SEM. **f-h** FlyPAD assay of flies when sd-L neurons were silenced with kir2.1. Flies were all starved for 24 h before assay. Both 0.25% and 0.4% agarose containing 10 mM sucrose. Cumulative sips numbers of sd-L-SS flies, n = 16 for each group (**f**). Cumulative sip numbers of UAS-kir2.1flies, n = 16 for each group (**g**). Cumulative sip numbers of sd-L-SS > UAS-kir2.1 flies, n = 15 for each group (**h**). For all analyses, statistical differences are represented as follows: ns, not significant, p > 0.05; *, p < 0.05; **, p < 0.01; ***, p < 0.001; ****, p < 0.0001.

Previous studies have revealed that the mechanoreceptor neurons on the fly labellum detect and assess the texture of food during feeding [12]. To identify the sensory structures and molecules which are indispensable for distinguishing fine food texture, we performed a candidate screening for mechanosensitive channel genes essential for sensation of tactile or proprioceptive information. Among these candidates, *tmc* is involved in the detection of tactile information from food [11], whereas *inactive* (*iav*) and *nanchung* (*nan*) sense vibration and proprioceptive stimuli in chordotonal organ (Cho) neurons [27,28]. When flies were allowed to choose between 0.25% and 0.4% agarose, we found that all the mutants tested here showed an impaired ability to discriminate fine difference of food hardness (Fig 2a). The flies lacking the *iav* gene even showed a reversed texture preference. We then compared the preference of *iav* mutant between 0.25% agarose and a range of other concentrations. These flies showed severe impairments in discriminating 0.3% to 0.5% agarose-containing food from 0.25% agarose-containing food but not to higher hardness (Fig 2b). This is strong evidence that *iav* is the mechanotransduction channel required in labellar mechanoreceptor neurons for fine food hardness detection.

### Single-dendritic labellum neurons discriminate substrates of different stiffness during feeding

We previously showed that *iav* labels “single-dendritic labellum” (sd-L) neurons which are required for detecting subtle stiffness differences during egg-laying [29]. Sd-L neurons are iav+/nan+/tmc- labial neurons whose axon arborizations occupy the dorsal anterior subesophageal zone (SEZ), while the tmc+ md-L neurons essential for stiffness detection during feeding [11,29] occupy ventral area of SEZ. Therefore, we speculated that sd-L neurons might also sense subtle stiffness difference during feeding. In order to specifically label sd-L neurons, we performed intersectional genetic labeling using the iav-Gal4 line in combination with different Gal80 lines [30]. We identified one Gal80 line, vGluT-Gal80 [31], which specifically blocked the expression of iav in sd-L neurons in the labellum (S2a and S2b Fig). Co-localization between iav-Gal4 and vGluT-QF further confirmed that these two genes were co-expressed in sd-L neurons (S2c Fig). We then generated an sd-L driver using split-Gal4 system [32], in which two Gal4 domains could be independently targeted to different cells and only cells that expressed both Gal4 components could reconstitute Gal4 activity. Therefore, we combined vGluT-AD and iav-DBD, two components of split-Gal4 system, on one chromosome and named this driver of sd-L stable split Gal4 (sd-L-SS). Sd-L-SS labelled sd-L neurons that located at the junction between the labellum and the haustellum and projected into SEZ (Fig 2d, S3a and S3c Fig), the same as previously reported [29]. Sd-L-SS had no expression in the wings or ovipositors (S3d and S3e Fig), but labelled a neuron on foreleg tarsus which projected to ventral nerve cord (VNC) (S3a and S3b Fig).

To investigate the role of sd-L neurons in detecting food hardness, we silenced them using the inward rectifying potassium channel Kir2.1 [33]. When given a choice between 0.25% and 0.4% agarose containing 10 mM sucrose, control flies preferred 0.4% agarose, while flies with sd-L neurons silenced failed to choose between the two hardness (Fig 2c). Furthermore, when we blocked sd-L neurons with tetanus toxin (TNT), flies also failed to choose the 0.4% agarose (S2d Fig). Similar results were observed in FlyPAD assay in which parental control flies took more sips when given 0.4% agarose (Fig 2f and 2g, and S4c–S4f Fig) while flies with sd-L neurons silenced showed no difference between 0.25% and 0.4% agarose (Fig 2h, S4a and S4b Fig). To test whether sd-L neurons could regulate sipping and PER in the absence of sugars, we used plain agarose of 0.25% and 0.4% in FlyPAD assay. Wild type *w*^*1118*^ flies took more sips when given 0.4% agarose even if there was no sucrose (S5a–S5d Fig), while flies with sd-L neurons silenced showed no difference between 0.25% and 0.4% plain agarose (S5e–S5h Fig).

We also found that md-L (*tmc* positive) neurons and *nan*-postive mechanosensory neurons affected the preference for 0.4% agarose (S2d Fig) by blocking them with TNT, respectively. The flies with *nan* neurons blocked showed a similar defect in texture preference, likely because *iav* and *nan* were co-expressed in sd-L neurons. It should be pointed out that, Nan and Iav were reported to function interdependently in mechanotransduction and fly sensory neurons [28]. Mutation of Nan gene caused the impaired expression of Iav protein [28]. Furthermore, they form heteromeric channels in the sensory neurons and *in vitro* [34]. It’s thus possible that Nan and Iav function as a complex in sd-L neurons and loss of either gene leads to significant impairments in mechanosensation. Given the spatial proximity between the projections of md-L neurons and sd-L neurons in SEZ, they might be able to integrate the texture information from the peripheral [29] (S2f and S2g Fig). The defects of *iav* mutant flies can be rescued by expressing *iav* wild-type cDNA in the sd-L neurons driven by R41E11-gal4 (S2e Fig), which could also label sd-L neurons [29]. These results together support the notion that sd-L neurons detect subtle stiffness differences during feeding and require the gene *iav*.

### Activation of sd-L neurons in labellum promoted feeding

We then wondered whether the direct activation of sd-L neurons had a positive impact on feeding behavior. To test this, we optogenetically activated sd-L neurons via a red-shifted channelrhodopsin, CsChrimson [35]. We found that *w*^*1118*^ male flies showed increased PER response with the increase of sucrose concentration, and 20 mM sucrose was a moderate concentration to induce reliable PER (S6a Fig). So we used 20 mM sucrose to test PER response during optogenetic activation. Remarkably, flies showed increased PER when sd-L neurons were activated by exposure to 1 mW/cm^2^ light (595nm) (Fig 2e), indicating the activation of sd-L neurons promoted flies feeding. To test the sugar concentration used in the two-way choice assay, we also tested 10mM sucrose stimulation. Flies also showed increased PER when sd-L neurons were activated (S6c and S6d Fig). Interestingly, activation of sd-L neurons alone optogenetically was insufficient to induce PER response. We used water stimuli as control during optogenetic activation before and after the sugar test, and almost no flies showed PER response to water with light on (S6b Fig).

Considering that there was a neuron labelled with sd-L-SS on the leg which projected to VNC (S3a and S3c Fig), we removed all the legs of flies and tested the PER response. To test the sugar concentration used in the two-way choice assay, we also tested 10mM sucrose stimulation. Flies showed increased PER to both 10mM and 20mM sucrose when sd-L neurons were activated, and only activation of sd-L on the labellum was sufficient to enhance the PER (S6c and S6d Fig). Taken together, activation of sd-L neurons in labellum can promote feeding on sugar-containing food.

### sd-L neurons activate sweet-sensing neurons to induce feeding preference

Flies evaluate the nutritional value of food by gustatory receptor neurons (GRNs), among which, the Gr64f/Gr5a sugar-sensing neurons mediate palatability of nutrient-rich food [36–39]. As sd-L neurons also project to the SEZ, we wondered whether axons of sd-L neurons could directly contact those of sweet neurons in the SEZ.

**Fig 3:**
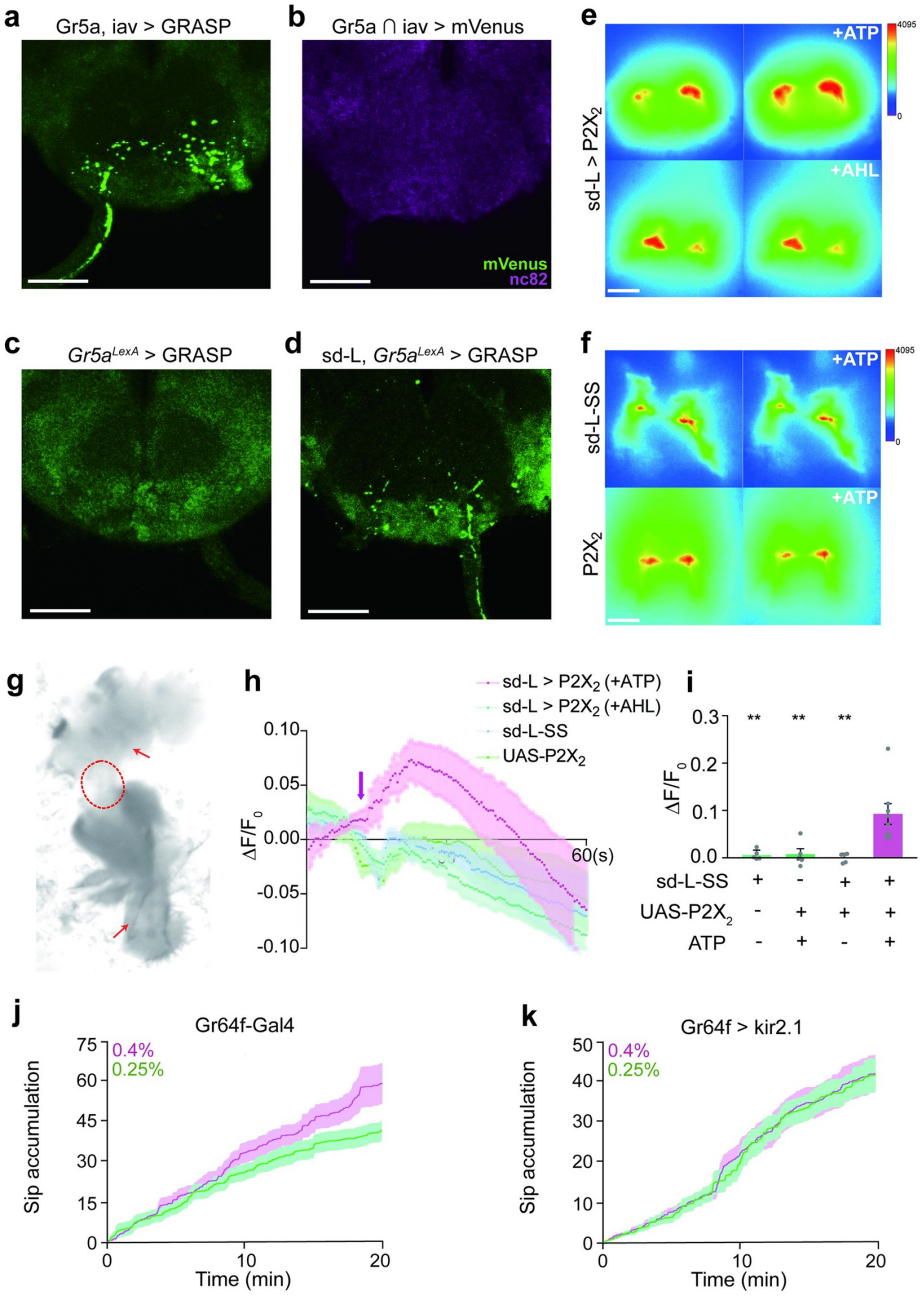
sd-L neurons signal to the sweet-sensing neurons to control hardness preference. **a** GRASP signal (green, anti-GFP) between iav-LexA and Gr5a-gal4 neurons (iav-LexA > lexAop-CD4-spGFP11 and Gr5a-gal4 > UAS-CD4-spGFP1-10) in the SEZ. Scale bar, 50 μm. **b** Co-localization signal (green, anti-GFP; magenta, anti-Brp) between iav-LexA and Gr5a-gal4 neurons (iav-LexA > 8×LexAop2-FLPL and Gr5a-gal4 > 20XUAS> dsFRT>chrimson-mVenus) in the SEZ. Scale bar, 50 μm. Magenta: nc82. **c, d** GRASP signal (green, anti-GFP) between *Gr5a*^*LexA*^ and sd-L neurons in the SEZ. (c) *Gr5a*^*LexA*^ > lexAop-CD4-spGFP11, UAS-CD4-spGFP1-10; (d) sd-L spilt gal4 > UAS-CD4-spGFP1-10, *Gr5a*^*LexA*^ > lexAop-CD4-spGFP11. Scale bar, 50 μm. **e-i** Calcium imaging of the sweet-sensing neurons in response to the activation of sd-L neurons. (g) An image of fly preparation for calcium imaging. Red arrow pointed to SEZ region (top) and labellum (bottom). Labellum and brain were connected (circled by a dotted line). Sweet neurons express GCaMP6m under the control of Gr64f > LexA. Representative imaging of Ca^2+^ responses in the SEZ before (left) and after (right) the application of ATP or AHL were shown. Sd-L-SS drove the expression of P2X_2_, with 5 mM ATP (upper panels) or equal volume of AHL (lower panels) (**e**); Scale bar, 50 μm. Parental control (sd-L-SS only (upper panels) or UAS-P2X_2_ only (lower panels)) were given 5 mM ATP (**f**); Scale bar, 50 μm. Changes of fluorescence intensity along the recording time window (**h**), the purple arrow pointed the time when ATP or AHL were added in. Summary of maximum calcium responses of Gr64f neurons to sd-L neurons’ activation and controls (**i**); n = 5, 5, 5 and 8 for each group. Statistical test: one-way ANOVA with Dunnett’s correction for multiple comparisons against the sd-L-SS > P2X_2_ with ATP group; mean ± SEM. **j, k** FlyPAD assay of fly feeding when Gr64f neurons were silenced with UAS-kir2.1. Flies were all starved for 24 h before assay. Both 0.25% and 0.4% agarose containing 10 mM sucrose. Cumulative sips numbers of Gr64f-Gal4 flies, n = 15 for each group (**j**). Cumulative sips numbers of Gr64f-Gal4 > UAS-kir2.1 flies, n = 15 for each group (**k**). For all analyses, statistical differences are represented as follows: ns, not significant, p > 0.05; *, p < 0.05; **, p < 0.01; ***, p < 0.001; ****, p < 0.0001.

We first tested the relationship between sd-L neurons and the sweet-sensing neurons. Using the GRASP technique [24] that targeted two halves of the GFP: CD4-spGFP1-10 and CD4-spGFP11 to the cell membranes of the iav neurons and Gr5a neurons, respectively. And we detected reconstituted GFP at areas targeted by axons of iav-Gal4 and Gr5a-LexA neurons in the SEZ (Fig 3a). To exclude the possibility of the co-expression of the two lines, we intersected the Gr5a-gal4 with the iav-LexA driver line using FLP-recombinase-mediated recombination [40] and found that these two lines did not co-express in the brain (Fig 3b). To further confirm that sd-L neurons synapsed on the sweet-sensing neurons, we expressed the two halves of split GFP again in either sweet GRNs using *Gr5a*^*LexA*^ or in sd-L neurons using sd-L-SS. As expected, GRASP signals were observed in the SEZ (Fig 3c and 3d), suggesting that sd-L neurons might have a potential synaptic connection with sweet-sensing neurons.

To determine whether the potential connections relay excitation from sd-L neurons to the sweet-sensing neurons, we monitored the Ca^2+^ influx at the axon termini of the sweet-sensing neurons with GCaMP6m [41] while stimulating sd-L neurons with the ATP-gated P2X_2_ channel in an *ex vivo* preparation (Fig 3g) [42,43]. We found that stimulating the P2X_2_-expressing sd-L neurons with ATP triggered a substantial increase of GCaMP signals in the axonal termini of the sweet-sensing neurons (Fig 3e, 3h and 3i). In contrast, stimulating sd-L neurons from the parental control flies did not elicit a detectable increase in those neurons (Fig 3f, 3h and 3i). These results suggested that activation of sd-L neurons enhanced the phagostimulatory effect of sugar food by increasing the presynaptic gain in the sweet GRNs.

Since sweet neurons received signals from sd-L neurons, are they involved in discrimination of different hardness? Using FlyPAD assay, we found that flies lost the ability to choose between 0.25% and 0.4% agarose after their sweet neurons were silenced (Fig 3k, S7a and S7b Fig) while both parental controls showed normal preference as wild flies (Figs 3j and 2g, S7c and S7d Fig). We have shown that both sugar stimulation and activation sd-L neurons were required to trigger PER (S6b Fig), and the sweet-sensing neurons which receive excitatory signals from sd-L neurons were also required for the discrimination of subtle difference of food hardness. Thus, there may be functional axo-axonal connections between sd-L neurons and sweet-sensing neurons. Alternatively, sd-L neurons may connect sweet-sensing neurons via yet unidentified interneurons. Taken together, we confirmed that mechanosensitive sd-L neurons promoted feeding preference of fine hardness by directly or indirectly enhancing the activity of the sweet-sensing neurons.

### Identification of the second-order neurons of sd-L neurons

Now we have demonstrated that sd-L neurons signal to the sweet-sensing neurons to promote the preference of hard food during feeding, which explains results of the two-way choice assay (Fig 1b). However, the flies’ haustellate mouthparts are adapted to suck liquid or sponge from liquefied food. We thus speculated that the activation of sd-L neurons would facilitate food ingestion.

**Fig 4:**
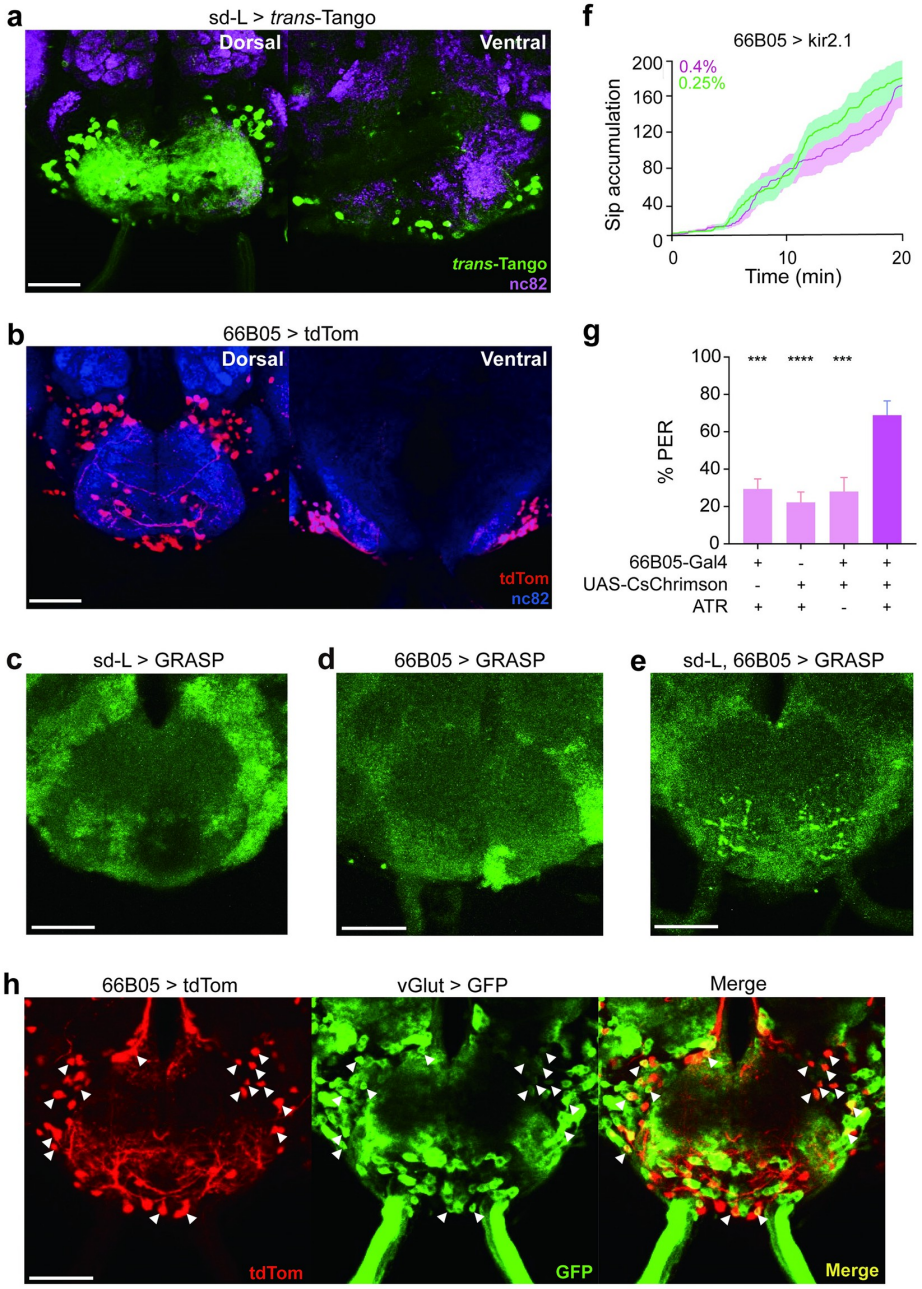
R66B05 labels a subset of the second-order neurons of sd-L neurons. **a** Putative second order neurons of sd-L neurons revealed by *trans*-Tango (sd-L spilt gal4 > UAS-*trans*-tango; green, anti-GFP; magenta, anti-Brp). Scale bar, 50 μm. Magenta: nc82. **b** R66B05-Gal4 drove the expression of tdTomato (R66B05-Gal4 > UAS-CD4-tdTomato; red, anti-RFP; bule, anti-Brp) in the SEZ. Scale bar, 50 μm. Blue: nc82. **c-e** GRASP signal (green, anti-GFP) between sd-L and R66B05-LexA neurons in the SEZ. (**c**) sd-L spilt gal4 > UAS-CD4-spGFP1-10, lexAop-CD4-spGFP11; (**d**) R66B05-LexA > UAS-CD4-spGFP1-10, lexAop-CD4-spGFP11; (**e**)sd-L spilt gal4 > UAS-CD4-spGFP1-10, R66B05-LexA > lexAop-CD4-spGFP11. Scale bar, 50 μm. **f** Cumulative sip numbers of R66B05-Gal4 > UAS-kir2.1 flies in FlyPAD assay. Flies were all starved for 24 h before assay. Both 0.25% and 0.4% agarose contained 10 mM sucrose, n = 14 and 16 for each group. **g** PER assay of flies when R66B05 neurons were activated by CsChrimson by exposure to 1 mW/cm^2^ light (595nm). Flies were tested with 20 mM sucrose. n = 15, 18, 15 and 18 for each group. Statistical test: one-way ANOVA with Dunnett’s correction for multiple comparisons against the R66B05-Gal4 > UAS-CsChrimson with ATR group; mean ± SEM. **h** Co-localization between R66B05-Gal4 > UAS-CD4-tdTomato (red, anti-RFP) and vGluT-QF > QUAS-mCD8-GFP (green, anti-GFP) in the brain. White arrow pointed to the overlapping neurons. Scale bar, 50 μm. For all analyses, statistical differences are represented as follows: ns, not significant, p > 0.05; *, p < 0.05; **, p < 0.01; ***, p < 0.001; ****, p < 0.0001.

We next explored how the texture information sensed by sd-L neurons was integrated into the feeding motor control circuit. We first used *trans*-Tango, a method for anterograde *trans*-synaptic tracing [44], to identify putative second-order neurons of sd-L neurons. In flies bearing the sd-L-SS driver and the *trans*-Tango components, we observed dozens of neurons with their cell bodies located in the SEZ (Fig 4a), indicating that sd-L neurons mainly target the feeding control center of the brain. Then we screened thirty-four fly lines that showed a similar expression pattern in the SEZ with the *trans*-Tango labeled neurons. We found a driver R66B05-Gal4 that appeared to label the second-order neurons of sd-L neurons (Fig 4b). To validate this, we used the GRASP technique and observed intense reconstituted GFP signals between sd-L neurons and R66B05-labeling neurons in the SEZ (Fig 4c–4e), suggesting that sd-L neurons and R66B05 neurons may form synapses.

To test whether R66B05 neurons were involved in food texture discrimination, we conducted FlyPAD assay. When R66B05 neurons were silenced, flies showed no feeding preference between 0.25% and 0.4% agarose (Fig 4f, S8a and S8b Fig), while parental control flies took more sips on 0.4% agarose than 0.25% agarose (Fig 2g and S8c–S8e Fig). Moreover, when R66B05 neurons were optogenetically activated, the flies showed higher PER percentage than their control lines, similar to what were observed in the sd-L neurons’ activation experiments (Figs 2e and 4g).

The above results suggest that R66B05 are downstream neurons of sd-L neurons. But how do they participate in the feeding control? There are two possible mechanisms: 1, R66B05 neurons are interneurons that integrate sensory inputs from the peripheral, including those from sd-L neurons. 2, R66B05 neurons are themselves motor neurons that can be activated by sd-L neurons and promote feeding action. To differentiate the two possibilities, we performed co-localization experiment between 66B05-Gal4 and vGluT-QF, a glutaminergic neuron driver to label motor neurons in *Drosophila* [31]. These two neuronal populations partially co-localized (Fig 4h), indicating that some of the R66B05 driver labelled neurons are motor neurons in the SEZ. Although we can’t exclude the existence of interneurons in the R66B05 driver labelled neurons, this echoes our speculation that sd-L neurons may be the upstream of some interneurons in SEZ.

### sd-L neurons synapse to subsets of motor neurons to control feeding

**Fig 5:**
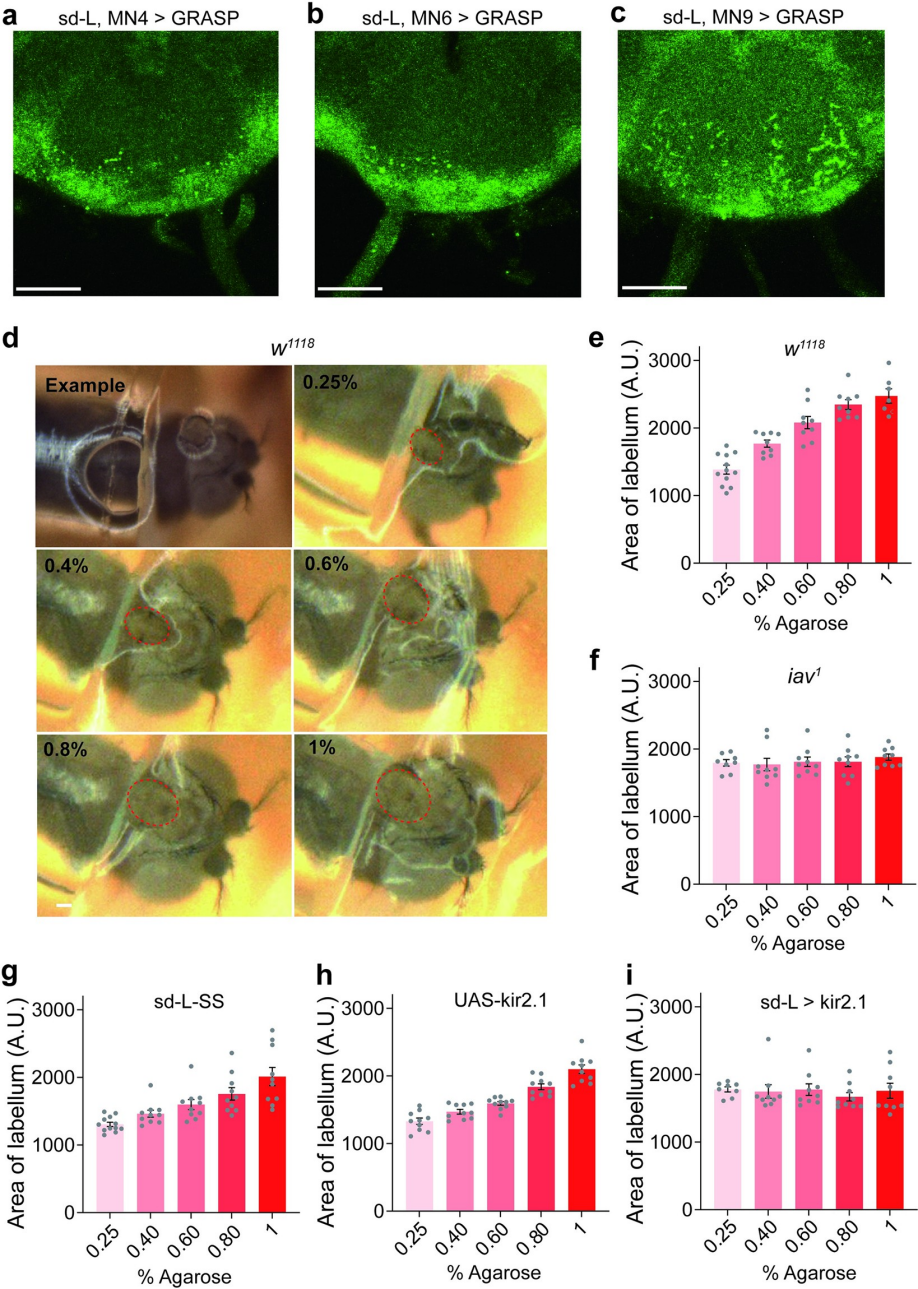
Sd-L neurons project axons to motor neurons to control feeding action. **a-c** GRASP signal (green, anti-GFP) between sd-L neurons and MN4 (**a**), MN6 (**b**) and MN9 (**c**) neurons in the SEZ. (**a**) sd-L spilt gal4 > UAS-CD4-spGFP1-10 and R45G01-LexA > lexAop-CD4-spGFP11. (**b**) sd-L spilt gal4 > UAS-CD4-spGFP1-10 and R81B12-LexA > lexAop-CD4-spGFP11. (**c**) sd-L spilt gal4 > UAS-CD4-spGFP1-10 and R18B07-LexA > lexAop-CD4-spGFP11. Scale bar, 50 μm. **d** The labella spreading of *w*^*1118*^ flies when feeding on 0.25%, 0.4%, 0.6%, 0.8% or 1% agarose. All concentrations of agarose containing 100 mM sucrose. The red circle outlined the labellum lobe area. Scale bar, 100 μm. **e-i** Quanficatioin of labellum spreading area of *w*^*1118*^ (**e**), *iav*^*1*^ (**f**), sd-L-SS (**g**), UAS-kir2.1 (**h**) and sd-L-SS > UAS-kir2.1 (**i**) flies when fed with 0.25%, 0.4%, 0.6%, 0.8% or 1% agarose containing 100 mM sucrose. Data are represented as mean ± SEM; n = 8~12 for each group.

Proboscis motoneurons are located in the SEZ and innervate muscle groups that potentially contributing to proboscis movement and food ingestion [45–47]. Upon a palatable gustatory stimulus, several groups of motor neurons control different steps of feeding, for examples, lifting the rostrum (MN9), extending the haustellum (MN4&9), extending the labellum (MN6), spreading the labella for food ingestion (MN9) [15,46,48], etc. Several driver lines were reported to label these MNs: MN9 are labelled by GMR18B07, MN4 are labelled by GMR45G01 and MN6 are labelled by GMR81B12 [15,46,48]. We then examined whether sd-L neurons formed synapses with these motor neurons. As expected, reconstituted GFP signals were found between sd-L neurons and these three MN types (Fig 5a–5c), suggesting that these motor neurons may receive inputs from sd-L neurons.

During feeding, flies spread their two labellar lobes immediately when the labella touched the food [15] and this action is controlled by different sets of motor neurons [15,45–47]. We then tested whether labellum-spreading during feeding was affected by food hardness. With the increase of agarose concentration, the labellum-spread area of *w*^*1118*^ flies gradually increased (Fig 5d and 5e), indicating that flies need to extend and spread their labellum to a greater extent when encountering harder food. However, *iav*-mutated or sd-L neurons-silenced flies showed no differences in labellum-spreading when feeding agarose of different concentrations (Fig 5f–5i). These results support the notion that sd-L neurons can access the feeding-promoting motor neurons through direct or indirect way to promote both the preference and the single-choice behavior between sugar-containing 0.4% agarose and sugar-containing 0.25% agarose.

### Mating promotes feeding on softer food via a sex peptide-dependent pathway

**Fig 6:**
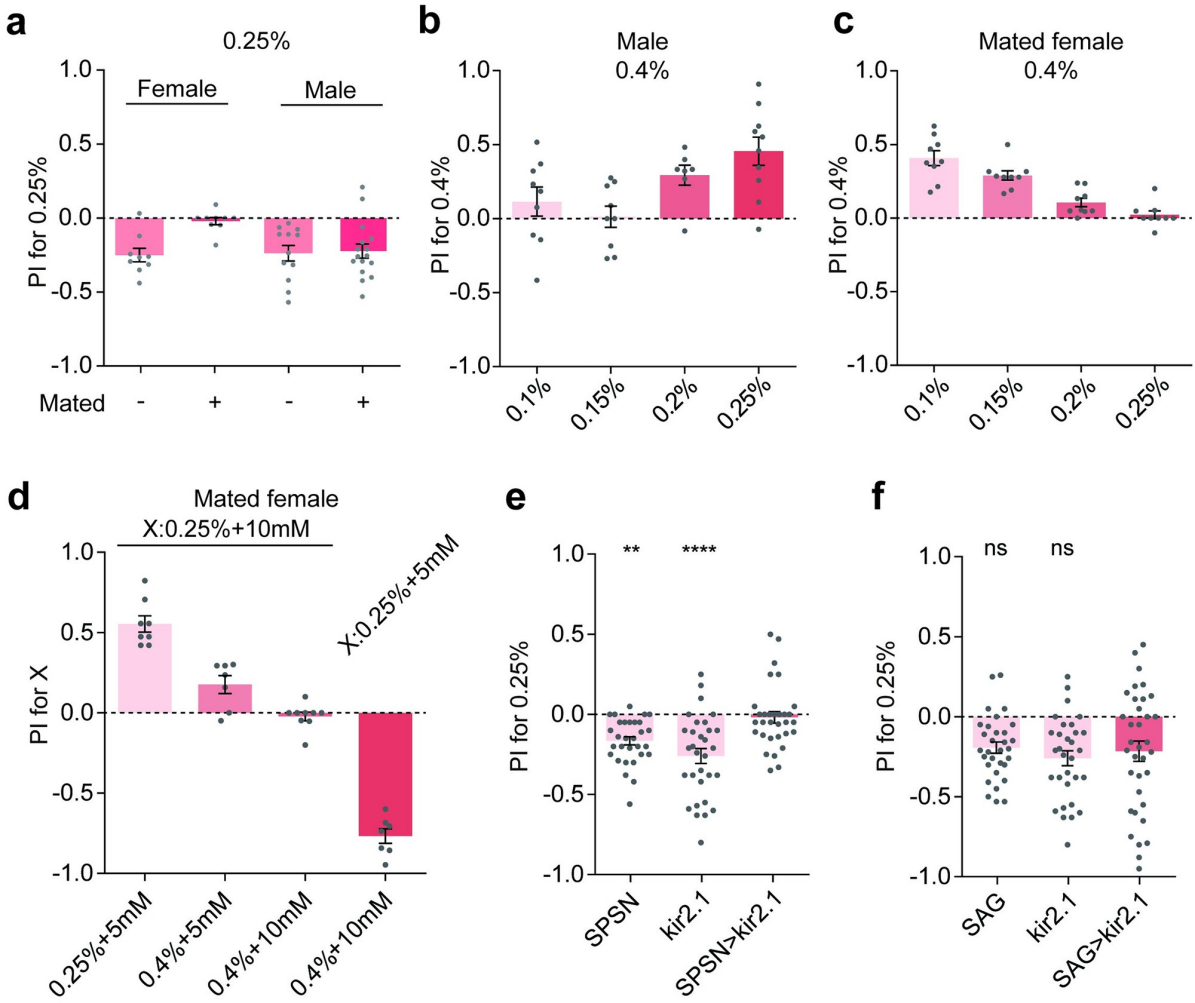
Mating promote feeding on softer food via a sex peptide pathway. **a** The preference of food hardness of *w*^*1118*^ males and females in the two-way choice feeding assay. “Mated+” means flies with mating experiences and “Mated-” means flies without any mating experience (virgin). Besides of Fig 6a, all males used in other experiments are “Mated-” males. 10 mM sucrose was added to both 0.25% and 0.4% agarose. Each gray point represents one independent trial and the number of points per bar indicates the number of replications in each experiment. n = 9, 9, 12, 16 for each group. Statistical test: unpaired Mann-Whitney test; mean ± SEM. **b, c** The preference of food hardness of *w*^*1118*^ males (**b**) and *w*^*1118*^ mated females (**c**) in the two-way choice feeding assay. PI for 0.4% of *w*^*1118*^ tested under 4 ranges of stiffness difference (0.1%, 0.15%, 0.2%, 0.25% vs 0.4%). 10 mM sucrose was added to different concentrations of agarose. n = 9 for each group; mean ± SEM. **d** The preference of hardness and sucrose of *w*^*1118*^ mated females in the two-way choice feeding assay. For the first three columns, X represents 0.25% agarose with 10mM sucrose; for the forth column, X represents 0.25% agarose with 5mM sucrose. The calculation of PI for X was the same as used for the calculation of PI for 0.25% in Fig 1a. n = 8, 7, 9 and 7 for each group; mean ± SEM. **e** The preference of food hardness when SPSN neurons (labeled by VT3280-Gal4) were silenced with Kir2.1 in the two-way choice feeding assay. Virgin females were used to choose between 0.25% and 0.4% agarose. n = 31, 30 and 31 for each group. Statistical test: one-way ANOVA with Dunnett’s correction for multiple comparisons against the VT3280-Gal4 > UAS-kir2.1 group; mean ± SEM. **f** The preference of food hardness when SAG neurons (labeled by two spilt-gal4: VT050405-p65.AD and VT007068-GAL4.DBD) were silenced with kir2.1 in the two-way choice feeding assay. Virgin females were used to choose between 0.25% and 0.4% agarose. 10 mM sucrose was added. n = 35, 31 and 31 for each group. Statistical test: one-way ANOVA with Dunnett’s correction for multiple comparisons against the VT050405-p65.AD; VT007068-GAL4.DBD > UAS-kir2.1 group; mean ± SEM. For all analyses, statistical differences are represented as follows: ns, not significant, p > 0.05; *, p < 0.05; **, p < 0.01; ***, p < 0.001; ****, p < 0.0001.

So far, we have shown that flies generally prefer to “chewy” food. We then asked whether this preference is regulated at different states. Softer food is usually more thoroughly fermented and are thus rich in yeast and polyamines, the nutrients that the mated females are strongly attracted to. With the two-choice assay, we observed a switch of food stiffness preference in the newly mating female flies. In contrast to virgin flies and male flies, mated ones showed a shifted PI for softer food when allowed to choose between 0.25% and 0.4% agarose (Fig 6a).

We found that male and virgin flies had the similar preference between 0.25% and 0.4% agarose (Fig 6a). As males were more convenient for us to observe the color of fly’s abdomen in the two-way choice assay, we selected males for subsequent experiments to better quantify the preference and reduce errors. This propensity to feed on softer food became stronger when food softer than 0.25% were given against the 0.4% agarose (Fig 6b and 6c). Interesting, when mated flies were allowed to choose between food with conflicting chemical and mechanical cues, e.g. harder/sweeter vs. softer/less sweeter food, they appeared to prefer the harder/sweeter one (Fig 6d), suggesting that the nutritional value of the food play a predominate role in making the final feeding decision.

We next sought to decipher the neural mechanisms underlying this regulation. The sex peptide and its downstream neural circuits mediate the diverse post-mating responses [21,49–52]. We first silenced the sex-peptide sensing neurons (SPSNs) with Kir2.1 that mimicked the sex-peptide induced inhibition of these neurons [21]. The flies showed no preference for harder food as seen in mated flies (Fig 6e), indicated the inhibition of SPSNs was sufficient to induce the change of food stiffness preference. However, when we silenced the SAG (SP abdominal ganglion) neurons, a downstream of SPSNs, the preference of harder food was not suppressed (Fig 6f), although silencing SAG neuron was effective to induce PMRs (post-mating responses) such as egg-laying [21,53]. Our current results suggest that the sex peptide acts on SPSNs to increase post-mating preference for softer food but the downstream circuit is distinct from the SAG pathway that was established to trigger egg-laying.

## Discussion

**Fig 7:**
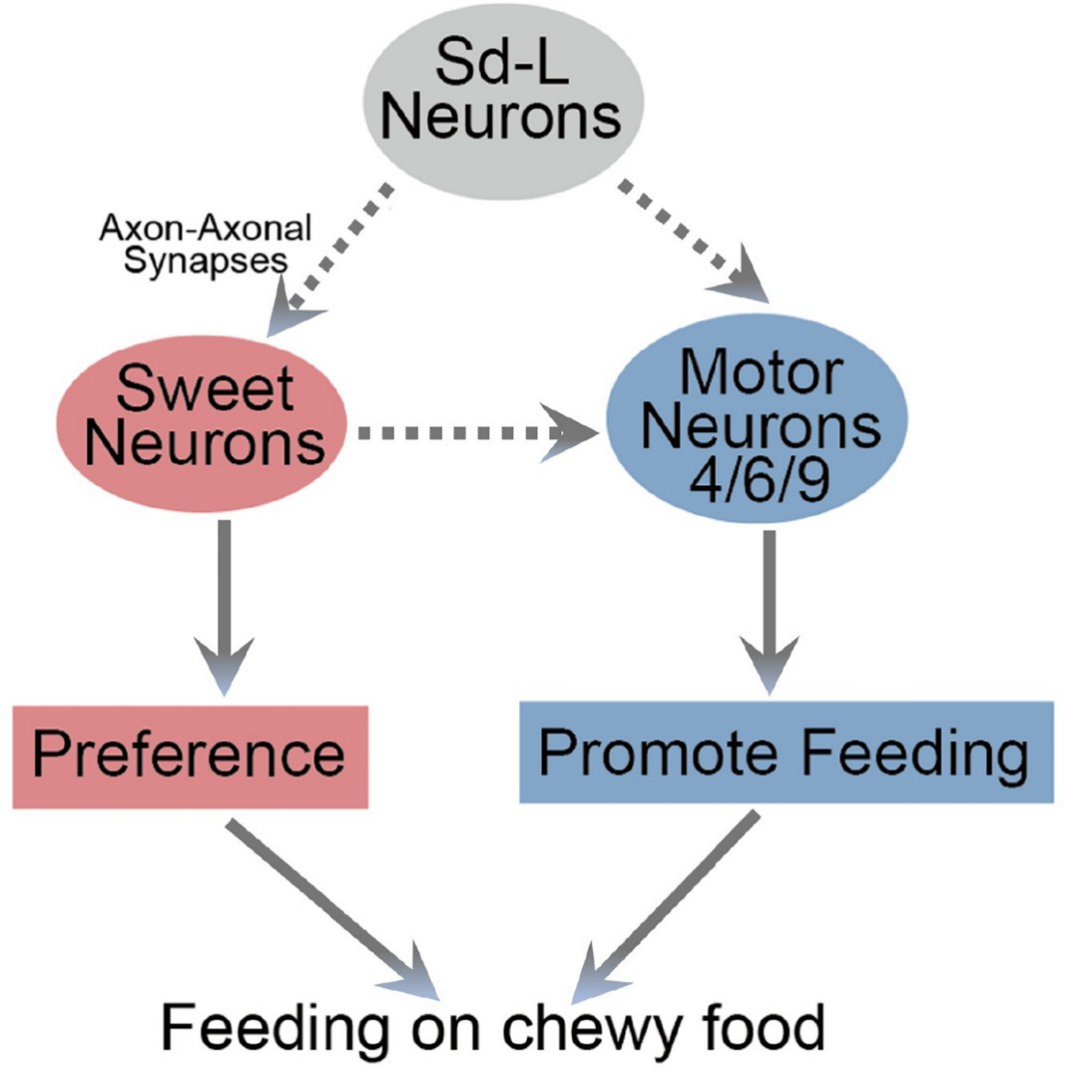
A working model on how sd-L neurons control fruit fly’ feeding on chewy food. sd-L neurons and the sweet-sensing neurons formed axo-axonal synapses to induce preference to harder food. At the same time, sd-L neurons connected with motor neurons 4/6/9 that innervating the labellum to coordinate the feeding actions. The connection between sd-L and sweet-sensing neurons or feeding-promoting motor neurons can be directly or indirectly (bridged by interneurons). These two neural circuits jointly control the feeding behavior on chewy food.

In this work, we have demonstrated that sd-L neurons not only signal to the sweet-sensing neurons to direct the selection for harder food, but also access the motor neurons innervating the labellum to coordinate the feeding action. In addition, sd-L neurons activate sweeting-sensing neurons to promote feeding, probably by regulating the activity of motor neuron (Fig 7).

### A preference for optimal food texture

Food with moderate stiffness maybe beneficial for animals. It’s generally thought that food that is too hard is less attractive. Here we have shown that food sources with too low stiffness are also less attractive to flies. Flies can discriminate hardness difference as small as 0.1% agarose to select the hardness around 0.4% agarose. This hardness is close to the fully ripe fruits such as banana or strawberries [12]. Food sources with hardness lower than this range could become watery and sticky and increase the risk that the flies get stuck when feeding on them. However, with a lapping mouthpart, flies would need squeeze harder to sponge the same amount of liquid from a harder food surface. To overcome this, sd-L neurons directly or indirectly synapse onto feeding-control motor neurons to exert more force so that flies can effectively ingest from a hard food source.

### Integration of texture information to the feeding circuit

The fly labellum is equipped with multiple mechanoreceptors to detect food texture. Among them, the NompC/Nan+ mechanosensory neurons underneath the taste sensilla, the Tmc+ md-L neurons and the NompC+ peg neurons all inhibit feeding [10–12,15]. The sd-L neurons are the first identified mechanosensory neurons that promote food ingestion. The sd-L neurons are located at the junction of the labellum and the haustellum, allowing them to sense the deformation of the proboscis during feeding. This sensory pathway works synergistically with the nanchung-expressing bipolar neurons to sense different range of stiffness. Iav functions in sd-L neurons but not in the nanchung-expressing bipolar neurons, thus the mutation of Iav only impairs the sense for fine texture. As sd-L neurons are activated by hardness about 0.3% agarose and beyond, they are likely activated by the hard substrates, e.g. agarose over 1% that suppress feeding. It thus plausible to hypothesize that the activation of sd-L neurons on sugar-sensing neurons are antagonized by the inhibitory inputs from labellum mechanosensory neurons onto the sugar-sensing neurons, so that the preference is limited to small range of food hardness around the optimal value.

### Multifaceted roles of sd-L neurons

The extensive arborizations in SEZ of sd-L neurons suggest that these neurons may form connections with multiple feeding control circuits. Here we have shown that sd-L neurons directly or indirectly signal gustatory receptor neurons and motor neurons. It’s tantalizing to hypothesize that they target certain interneurons in SEZ based on two observations: 1, The synapses between sd-L and sensory/motor neurons only occupy a small region of their axonal projections. 2, The second order neurons of sd-L labelled by *trans*-tango include some neurons that are local to SEZ. The function of sd-L in directing oviposition sites further support this notion [29]. However, it warrants further investigation how sd-L impart different tones on feeding selection and egg-laying decision.

### A divergent regulation for post-mating response

SP acts on neurons and non-neuronal tissues [49,50]. The neural pathway that mediated the sex peptide elicited egg-laying is the SPSNs-SAG-PC1 circuits [21,51–55]. In contrast, the switch on diet is mediated by a direct or indirect regulation of the chemosensory receptors and their downstream circuits [17,20]. Here we found that silencing SPSNs but not SAG was sufficient to induced the switch for food stiffness preference, suggesting the SPSNs may target downstream neurons other than SAGs/MIP neurons. Additionally, as MIP is release upon mating and the peptide targets the SP receptor [56], it’s conceivable to hypothesize the MIP directly modulate sd-L neurons or their downstream neural circuit to induce the change on food stiffness preference, as seen in the post-mating switch for diet.

### Animals

Fruit fly *Drosophila melanogaster* strains were kept with a 12 h/12 h light/dark cycle at 25°C and 60% humidity (PERCIVAL incubator). All flies were raised on standard cornmeal medium, which consisted of 10 g agar, 7.25 g sucrose, 30 g glucose, 24.5 g yeast, 50 g corn meal, 17.5 ml methyl 4-hydroxybenzoate and 4 ml propionic acid per 1L. Inside-lab breeding *w*^*1118*^ strain (BDSC:5905) was used as wild-type control.

Transgenic lines from Bloomington *Drosophila* Stock Center (BDSC) included: *w*^*1118*^ (5905), iav-Gal4 (52273), iav-LexA::p65 (52246), vGluT-QF, QUAS-mCD8-GFP (60315), *nan*^*Gal4*^ (68205), *tmc*^*1*^ (66556), tmc-GAL4 (66557), Gr5a-Gal4 (57592), R66B05-Gal4 (39389), R66B05-LexA (54917), *Gr33a*^*Gal4*^ (31425), LexAop-GCaMP6m (44590), 20XUAS-IVS-CsChrimson.mVenus (55135), 8XLexAop2-FLPL (55820), UAS-CD4-tdTomato (III) (35837), UAS-CD4-tdTomato (II) (35841), 10XUAS-IVS-mCD8::GFP (II) (32186), 10XUAS-IVS-mCD8::GFP (III) (32185), UAS-*trans*-tango (77123), UAS-CD4-spGFP1-10; LexAop-CD4-spGFP11 (58755), UAS-CsChrimson (55136), UAS-mCherry.NLS (38424), R18B07-LexA (52526), R45G01-LexA (54866), R81B12-LexA (54389), VT050405-p65.AD; VT007068-GAL4.DBD (66875). iav-DBD was generated by Dr. Liwei Zhang from Dr. Wei Zhang lab. *iav*^*1*^ was kindly provided by Dr. Yuh-Nung Jan at UCSF and vGluT-gal80 was kindly provided by Dr. Yi Zhong at THU. We thank Dr. Yanmeng Guo at UCSF for kindly providing *tmc-s*^*Gal4*^ strain, Dr. Hubert Amrein at Texas A&M University for kindly providing *Gr5a*^*LexA*^ strain and Dr. Barry J. Dickson at Howard Hughes Medical Institute for kindly providing VT3280-Gal4. UAS-P2X_2_ was a gift from Dr. Yufeng Pan at Southeast University, China and vGluT-AD was a gift from Dr. Yi Rao at Peking University, China. More detailed information of fly strains were listed in Table 1.

## Method details

### Two-way choice feeding assay

The two-way choice feeding assay was performed as previously described [57]. Briefly, 3~5 days old male flies were starved in an empty vial with a small piece of filter paper soaked with distilled water for 24 hours. The flies were then transferred to plastic Petri dishes with four-quadrant dividers (diameter: 90 mm) that were used to establish differential stiffnesses with indicated agarose concentrations. Every quadrant was loaded with 5 mL agarose mixed with either blue (food blue NO.1, 0.1 mg/ml) or red food dye (food red NO.106 0.1 mg/ml). Unless otherwise noted, 10 mM sucrose was added to different concentrations of agarose. The filled Petri dishes were allowed to solidify for 30 min at RT. Naïve males were anaesthetized on a CO_2_ pad and 30 fruit flies were introduced immediately into one Petri dish to feed freely for 1.5 hours in a dark box.

For feeding assay of females, 3~5 days old wild type virgin females and males were kept in groups. For mated females group, 20 virgins were mated with 23~25 males in food vials for 3 hours. Then females were separated on a CO2 pad and introduced immediately into the Petri dish to feed freely for 24 hours in a dark box. For virgin group, 20 virgins were introduced directly into the Petri dish to feed freely for 24 hours in a dark box. For mated males group, 20 males were mated with 25~30 virgin females in food vials for 3 days. Then males were separated on a CO2 pad and introduced immediately into an empty vial with a small piece of filter paper soaked with distilled water for 24 hours. Preference index was defined as the feeding preference for 0.25% agarose (reference stiffness) over the other agarose concentrations and calculated using the following equation: (N_0.25% agarose_−N_x% agarose_) / N_total colored flies_, in which X represents the agarose concentration in the neighboring quadrants of the 0.25% agarose sites. The number of flies was counted with the color of their abdomens showing red (N_red_) or blue (N_blue_) or mixed purple (N_purple_) under a stereomicroscope.

### Proboscis extension response (PER) assay

The proboscis extension response (PER) assay was performed as previously described [58]. Newly-eclosed male flies were transferred into vials covered with aluminum foil, which contained standard food medium or standard food medium added with 1mM all-*trans* retinal (ATR), and fed for 4~6 days. Flies were starved in an empty vial with a small piece of filter paper soaked with distilled water for 24 hours before the experiment. To test the PER, a fly was gently introduced into a 200 μl pipette tip with the head exposed. 100 mM sucrose solution and double distilled water were given to the flies as positive and negative control before and after the test, respectively. A drop of 20 mM sucrose solution was applied to the labellum with 30 s interval under light activation (595 nm). The PER% was counted as the proboscis extension times out of 5 trials. Data were discarded if the fly responded to water or did not respond to 100 mM sucrose solution. For sd-l > CsChrimson activation, the light intensity was 1 mW/cm^2^; and for R66B05 > CsChrimson activation, the light intensity was 9 mW/cm^2^. For amputation experiments, all the legs were cut on a CO_2_ pad using fine surgical scissors (F.S.T 91500–09), and then starved in an empty vial with a small piece of filter paper soaked with distilled water for 24 hours before the PER assay.

### FlyPAD assay

FlyPAD (Fly Proboscis and Activity Detector) assay was carried out as previously described [59] with slight modifications. Flies were collected upon eclosion and aged for 3~6 days on standard cornmeal medium. For food deprivation, female flies were kept in an empty vial with filter paper soaked with water for 24 hours. Each fly was transferred to one feeding chamber on the FlyPAD board and recorded for 20 min. Sips were detected with Python codes (Cap2Sip) translated from the MATLAB codes [59,60]. Cumulative sips duration and accumulation were calculated as the summation of all sips detected along the 20 min time window. The codes we used are available at GITHUB: https://github.com/EBGU/Cap2Sip.

### Measurement of labellum spreading

The different concentration agarose with 100 mM sucrose 500 μL was spread on a glass slide and allowed to solidify for 10 min at RT to form an even layer of agarose. Naïve 3~5 days male flies were gently introduced into a 200 μL pipette tip with only the head exposed to ensure the proboscis full extension. The pipette tip was then placed on clay to keep the labellum upwards under a stereomicroscope. The glass slide with agarose was approached to the labellum of fruit fly until it touched the labellum. The position was kept for 20 s and repeated 3 times for each fruit fly. This process was recorded with a Basler acA640-90gc camera. Three random frames were captured and measured of each fruit fly using Fiji and the average was taken as the area of labellum spreading.

### Immunohistochemistry and confocal imaging

3~6 days adult flies were used for dissection in 0.015% Triton X-100 in 1 × PBS. Dissected brains were fixed in 4% paraformaldehyde (PFA, Cat#AR-0211, Dingguo Biotech, China) at RT for 20 min on a shaker. They were then washed with PBS and blocked in blocking buffer (1 × Normal Goat Serum in wash buffer) for 30 min at RT. After blocking, samples were incubated in primary antibodies in blocking buffer overnight on a shaker at 4°C. They were washed and incubated in secondary antibodies in blocking buffer for 2 hours on a shaker at RT. All washes were performed with 0.3% Triton X-100 in 1 × PBS for 3 × 20 min at RT. Primary antibody: rabbit anti-GFP (1:500, A11122, Invitrogen), mouse monoclonal nc82 (1:500, Developmental Studies Hybridoma Bank), anti-RFP (1:500, 600-401-379S, Rockland) and mouse monoclonal anti-GFP (1:500, G6539, Sigma). Secondary antibody: 488-goat anti-rabbit (1:200, A11008, Invitrogen), 647-goat anti-mouse (1:200, A21235, Invitrogen) and 555-goat anti-rabbit (1:200, A21428, Invitrogen). Imaging was performed on an Olympus FV1000 confocal microscope with 2.5 or 3 μm optical sections at a resolution of 1024 × 1024 pixels for labellum or 1024 × 800 pixels for brain. All images were processed with ImageJ software. More detailed information of antibodies were listed in Table 1.

### Calcium imaging

After brief ice anaesthesia, the head of 5~7 days old fly was immobilized in an imaging chamber with strips of double-sided sticky tape and the proboscis was kept extended (Fig 3g). The chamber was filled with artificial hemolymph -like solution (AHL): 103 mM NaCl, 3 mM KCl, 5 mM TES, 10 mM trehalose, 10 mM glucose, 26 mM NaHCO_3_, 1 mM NaH_2_PO_4_, and 4 mM MgCl_2_, pH 7.25, 310 mOsm). 2 mM CaCl_2_ was added to the saline before use [10, 61]. The antenna, compound eyes, brain cuticle and connective tissue covering the SEZ were removed using fine forceps. We made sure the connection between brain and labellum were intact, so that the activation of sd-L neurons can be relayed to the SEZ region. Calcium imaging was performed using an Olympus BX51WI microscope with a 40X water immersion objective, an Andor Zyla camera and a Uniblitz shutter. The images were acquired at 2 frame per second with a resolution of 512 × 512 pixels. The Ca^2+^ indicator GCaMP6m were used to measure the Ca^2+^ signal fluorescent signals were acquired from 20 s prior to ATP application with a final concentration of 2.5 mM to at least 60 s after the maximum fluorescence intensity. Ca^2+^ signal was collected prior to, during and following the application of ATP. For quantification, the fluorescence intensity of ROI was collected and plotted by ImageJ. The relative fluorescence change ΔF/F_0_ was calculated as following: ΔF/F_0_ = ((Max single frame intensity)—(Average intensity of 10 s right before ATP application (F_0_))) / (Average intensity of 10 s right before ATP application (F_0_)).

### Statistical analysis

GraphPad Prism 7 software was used to graph and statistically analyzed data. All datasets were presented as mean ± SEM. We used the two-tailed unpaired Student’s t test or one-way ANOVA followed with Dunnett’s multiple comparisons to analyze data from two-way feeding assay, PER assay and calcium imaging (see indications in each figure legend). For all analyses, statistical notations are as follows: ns, not significant, p > 0.05; *, p < 0.05; **, p < 0.01; ***, p < 0.001; ****, p < 0.0001. Each gray dot in the plotting represented an independent trial and the number of points per bar indicated number of replications (n) in each experiment. No sample size estimation and inclusion and exclusion of any data or subjects were conducted in this study.

## Supporting information

S1 Fig The experimental diagram for two-way choice feeding assay. (a) The preference for 0.25% agarose after dyes were switched. 5 mM sucrose was added to both 0.25% and 0.4% agarose-contained group. Dyes were switched and *w*^*1118*^ males were tested. n = 9 for both group; mean ± SEM. (b) The preference for food hardness of *w*^*1118*^ males in the two-way choice feeding assay. PI for 0.25% of *w*^*1118*^ males tested under 5 concentrations of stiffness (0.25% vs 0.25% ~ 0.5%). 5 mM sucrose was added to different concentrations of agarose. Each gray point represents one independent trial and the number of points per bar indicates the number of replications in each experiment. n = 10, 8, 10, 8 and 8 for each group; mean ± SEM.
(TIF)

S2 Fig Sd-L neurons are labelled by both iav and vGluT, and they are necessary for fine texture sensing. (a) Iav-Gal4 drove expression of tdTomato (red, anti-RFP) in the labellum (iav-gal4 > UAS- tdTomato). Scale bar, 50 μm. (b) Iav-Gal4 drove expression of tdTomato (red, anti-RFP) under the restriction of vGluT-Gal80 in the labellum (iav-gal4, vGluT-gal80 > UAS- tdTomato). Scale bar, 50 μm. (c) Co-localization between vGluT-QF (vGluT-QF > QUAS-mCD8-GFP, green, anti-GFP) and iav-Gal4 (iav-Gal4 > UAS-CD4-tdTomato, red, anti-RFP) in the labellum. White arrow pointed to sd-L neurons. Scale bar, 50 μm. (d) The preference of food hardness when sd-L/nan/tmc neurons were inhibited by TNT in the two-way choice feeding assay. 10 mM sucrose was added to different concentrations of agarose. Statistical test: one-way ANOVA with Dunnett’s correction for multiple comparisons against the sd-L-SS/nan-gal4/tmc-gal4 > UAS-TNT group; mean ± SEM; n = 11~19 for each group. (e) The rescue for the defects of *iav* mutant by expressing iav wild-type cDNA in the sd-L neurons. Sd-L neurons were labeled by R41E11-gal4. Statistical test: one-way ANOVA with Dunnett’s correction for multiple comparisons against the R41E11-gal4 > UAS-TNT group; mean ± SEM; n = 27, 29, 19, 36 for each group. (f) GRASP signal (green, anti-GFP) between iav-LexA and TMC-gal4 neurons (iav-LexA > lexAop-CD4-spGFP11 and TMC-gal4 > UAS-CD4-spGFP1-10) in the SEZ. Scale bar, 50 μm. (g) Co-localization signal (green, anti-GFP) between iav-LexA and TMC-gal4 neurons (iav-LexA > 8×LexAop2-FLPL and TMC-gal4> 20xU>dsFRT>chrimson-mVenus) in the SEZ. Scale bar, 50 μm. Magenta: nc82.
(TIF)

S3 Fig The expression pattern of sd-L neurons and connections between sd-L and the sweet-sensing neurons. (a) Expression patterns for sd-L-SS in the brain and the VNC. Immunostaining used either anti-GFP (green) and anti-Brp (blue). Genotype: sd-L-SS > UAS-GFP. Scale bar, 20 μm. (b-e) Expression patterns for sd-L-SS in the leg (b), labellum (c), wings (d) and ovipositors (e). Immunostaining used either anti-GFP. Genotype: sd-L-SS > UAS-GFP. Scale bar, 20 μm. (b) Red arrow pointed to one positive cell. (c) Red circle pointed to sd-L neurons and its dendrite.
(TIF)

S4 Fig Control flies of sd-L > kir2.1 consumed more 0.4% agarose than 0.25% on the FlyPAD. (a, b) FlyPAD assay of sd-L-SS > UAS-kir2.1 flies. Each vertical bar represents a single sip. Flies were feeding with 10 mM sucrose mixed with 0.25% agarose (a) or 0.4% agarose (b). n = 16 for each group. (c, d) FlyPAD assay of sd-L-SS (vGluT-AD; iav-DBD) flies. Each vertical bar represents a single sip. Flies were feeding with 10 mM sucrose mixed with 0.25% agarose (c) or 0.4% agarose (d). n = 16 for each group. (e, f) FlyPAD assay of UAS-kir2.1flies. Each vertical bar represents a single sip. Flies were feeding with 10mM sucrose mixed with 0.25% agarose (e) or 0.4% agarose (f). n = 16 for each group.
(TIF)

S5 Fig FlyPAD assay for feeding on plain agarose. (a-d) FlyPAD assay of *w*^*1118*^ male flies. Each vertical bar represents a single sip (a, b). Flies were fed with plain agarose of 0.25% (a) and 0.4% (b). Cumulative sip durations on 0.25% or 0.4% agarose (c). Cumulative sip numbers on 0.25% or 0.4% agarose (d). n = 22 for each group. (e-h) FlyPAD assay of sd-L-SS > UAS-kir2.1 male flies. Each vertical bar represents a single sip (e, f). Flies were fed with plain agarose of 0.25% agarose (e) and 0.4% agarose (f). Cumulative sip durations on 0.25% or 0.4% agarose (g). Cumulative sip numbers on 0.25% or 0.4% agarose (h). n = 16 for each group.
(TIF)

S6 Fig Activation of sd-L neurons in the labellum can promote feeding on sugar-containing food. (a) PER assay of *w*^*1118*^ male flies with the increase of sucrose concentration. 8 different sucrose concentrations (0 ~ 100 mM) were used to induce PER response. Data are represented as mean ± SEM; n = 10 for each group. (b) PER response to water during optogenetic activation of sd-L neurons. We counted all the flies’ water response before and after the sugar test, n = 66, 108, 62 and 180 for each group. (c-d) PER assay of flies when sd-L neurons were activated using CsChrimson by exposure to 1 mW/cm^2^ light (595nm). All the legs were removed and tested with 10 mM sucrose (c) or 20 mM sucrose (d). Statistical test: one-way ANOVA with Dunnett’s correction for multiple comparisons against the sd-L-SS > UAS-CsChrimson with ATR group; mean ± SEM; n = 10 ~ 15 for each group.
(TIF)

S7 Fig Control flies of Gr64f > kir2.1 consume more 0.4% agarose than 0.25% on the FlyPAD. (a, b) FlyPAD assay of Gr64f-Gal4 > UAS-kir2.1 flies. Each vertical bar represents a single sip. Flies were fed with 10 mM sucrose mixed with (a) 0.25% agarose or (b) 0.4% agarose. n = 16 for each group. (c, d) FlyPAD assay of Gr64f-Gal4 flies. Each vertical bar represents a single sip. Flies were fed with 10 mM sucrose mixed with (c) 0.25% agarose or (d) 0.4% agarose. n = 16 for each group.
(TIF)

S8 Fig Control flies of R66B05 > kir2.1 consume more 0.4% agarose than 0.25% on the FlyPAD. (a, b) FlyPAD assay of R66B05-Gal4 > UAS-kir2.1 flies. Each vertical bar represents a single sip. Flies were fed with 10 mM sucrose mixed with (a) 0.25% agarose or (b) 0.4% agarose. n = 14~16 for each group. (c, d) FlyPAD assay of R66B05-Gal4 flies. Each vertical bar represents a single sip. Flies were fed with 10 mM sucrose mixed with (c) 0.25% agarose or (d) 0.4% agarose. n = 16 for each group. (e) Cumulative sip numbers of R66B05-Gal4 flies in FlyPAD assay. Both 0.25% and 0.4% agarose containing 10mM sucrose, n = 16 for each group.
(TIF)

S9 Fig Quantification of labellum spreading area in different mutant flies. (a) Quantification of labellum spreading area of *nan*^*gal4*^ and *tmc*^*1*^ when fed with 0.25% or 0.4% agarose containing 100 mM sucrose. Data are represented as mean ± SEM; n = 10 ~ 11 for each group.
(TIF)
