# Supplementary figures and images for: A dedicate sensorimotor circuit enables fine texture discrimination by active touch
Source: PLoS Genet. 2023 Jan 17;19(1):e1010562. doi: 10.1371/journal.pgen.1010562 (PMC9882754; doi:10.1371/journal.pgen.1010562)

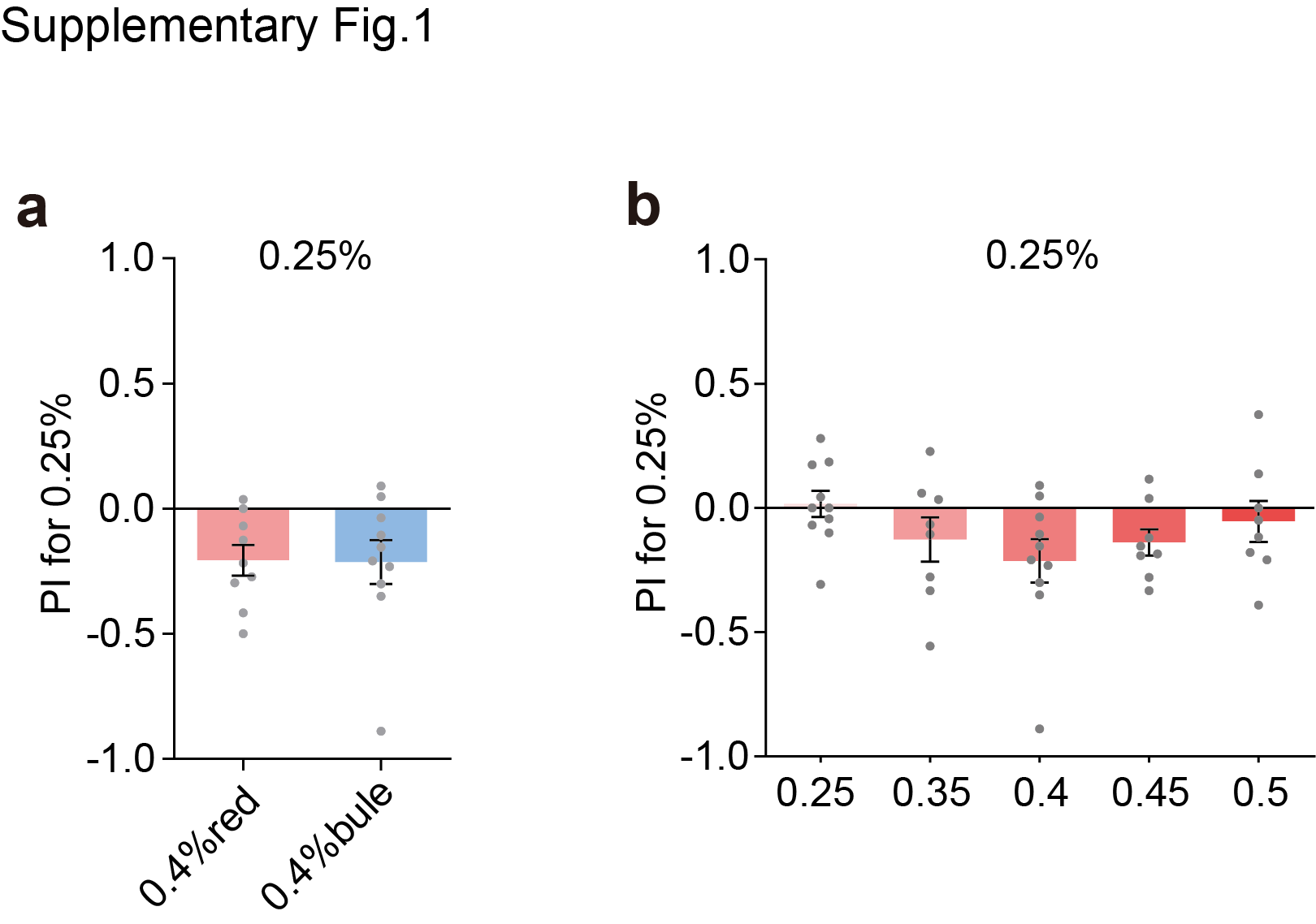

Supplement: S1 Fig — (a) The preference for 0.25% agarose after dyes were switched. 5 mM sucrose was added to both 0.25% and 0.4% agarose-contained group. Dyes were switched and w1118 males were tested. n = 9 for both group; mean ± SEM. (b) The preference for food hardness of w1118 males in the two-way choice feeding assay. PI for 0.25% of w1118 males tested under 5 concentrations of stiffness (0.25% vs 0.25% ~ 0.5%). 5 mM sucrose was added to different concentrations of agarose. Each gray point represents one independent trial and the number of points per bar indicates the number of replications in each experiment. n = 10, 8, 10, 8 and 8 for each group; mean ± SEM. (TIF) [file pgen.1010562.s001.tif]

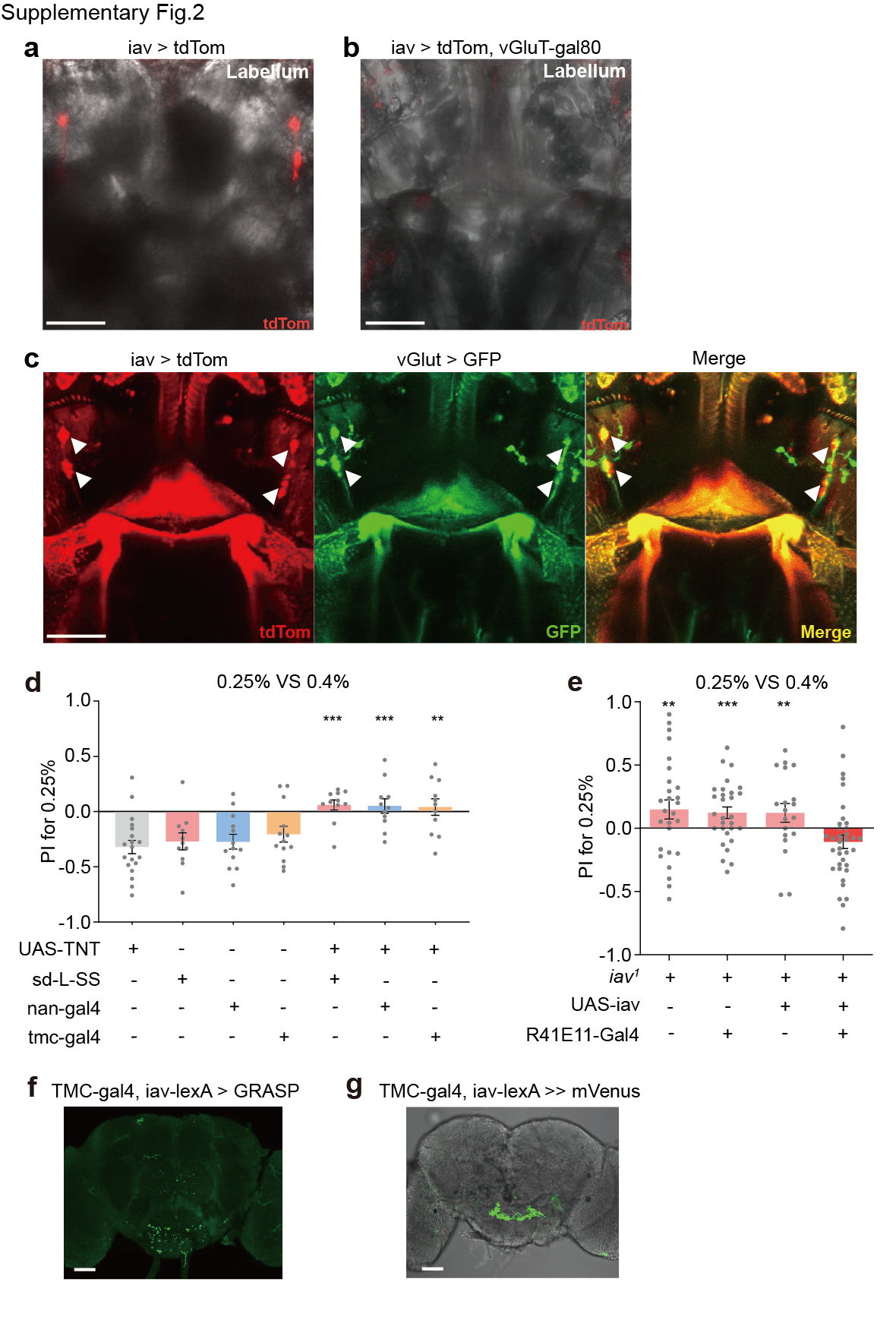

Supplement: S2 Fig — (a) Iav-Gal4 drove expression of tdTomato (red, anti-RFP) in the labellum (iav-gal4 > UAS- tdTomato). Scale bar, 50 μm. (b) Iav-Gal4 drove expression of tdTomato (red, anti-RFP) under the restriction of vGluT-Gal80 in the labellum (iav-gal4, vGluT-gal80 > UAS- tdTomato). Scale bar, 50 μm. (c) Co-localization between vGluT-QF (vGluT-QF > QUAS-mCD8-GFP, green, anti-GFP) and iav-Gal4 (iav-Gal4 > UAS-CD4-tdTomato, red, anti-RFP) in the labellum. White arrow pointed to sd-L neurons. Scale bar, 50 μm. (d) The preference of food hardness when sd-L/nan/tmc neurons were inhibited by TNT in the two-way choice feeding assay. 10 mM sucrose was added to different concentrations of agarose. Statistical test: one-way ANOVA with Dunnett’s correction for multiple comparisons against the sd-L-SS/nan-gal4/tmc-gal4 > UAS-TNT group; mean ± SEM; n = 11~19 for each group. (e) The rescue for the defects of iav mutant by expressing iav wild-type cDNA in the sd-L neurons. Sd-L neurons were labeled by R41E11-gal4. Statistical test: one-way ANOVA with Dunnett’s correction for multiple comparisons against the R41E11-gal4 > UAS-TNT group; mean ± SEM; n = 27, 29, 19, 36 for each group. (f) GRASP signal (green, anti-GFP) between iav-LexA and TMC-gal4 neurons (iav-LexA > lexAop-CD4-spGFP11 and TMC-gal4 > UAS-CD4-spGFP1-10) in the SEZ. Scale bar, 50 μm. (g) Co-localization signal (green, anti-GFP) between iav-LexA and TMC-gal4 neurons (iav-LexA > 8×LexAop2-FLPL and TMC-gal4> 20xU>dsFRT>chrimson-mVenus) in the SEZ. Scale bar, 50 μm. Magenta: nc82. (TIF) [file pgen.1010562.s002.tif]

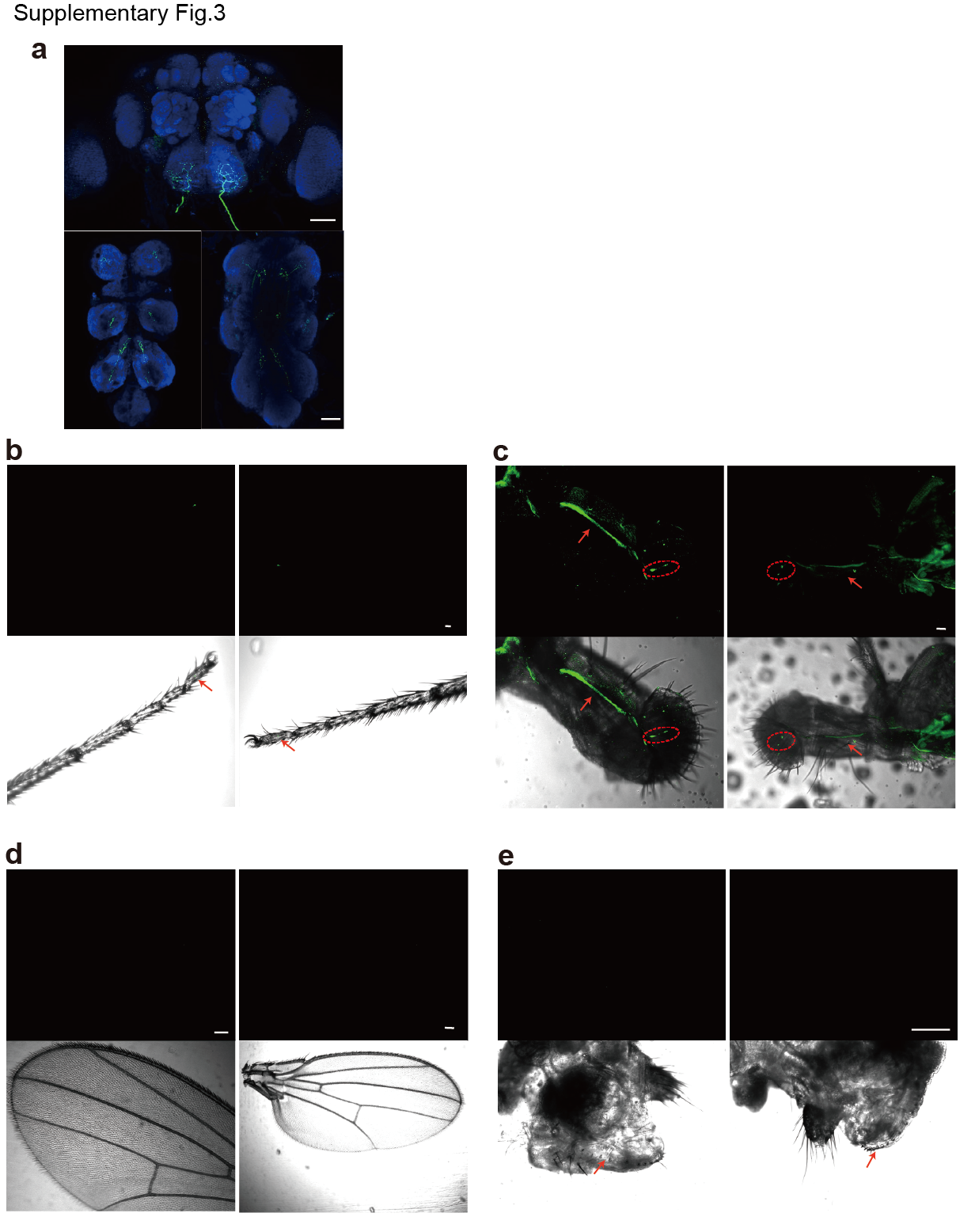

Supplement: S3 Fig — (a) Expression patterns for sd-L-SS in the brain and the VNC. Immunostaining used either anti-GFP (green) and anti-Brp (blue). Genotype: sd-L-SS > UAS-GFP. Scale bar, 20 μm. (b-e) Expression patterns for sd-L-SS in the leg (b), labellum (c), wings (d) and ovipositors (e). Immunostaining used either anti-GFP. Genotype: sd-L-SS > UAS-GFP. Scale bar, 20 μm. (b) Red arrow pointed to one positive cell. (c) Red circle pointed to sd-L neurons and its dendrite. (TIF) [file pgen.1010562.s003.tif]

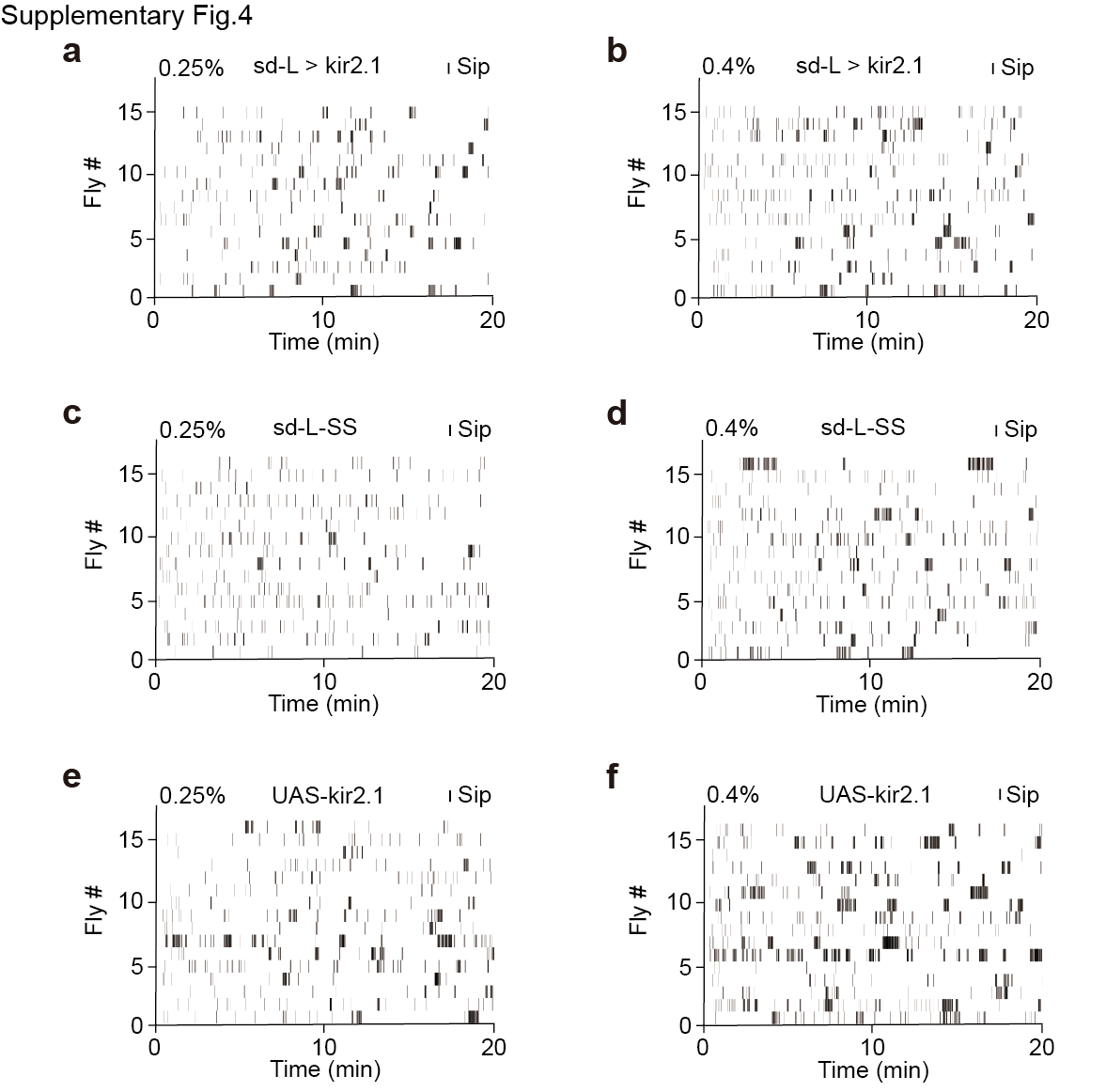

Supplement: S4 Fig — (a, b) FlyPAD assay of sd-L-SS > UAS-kir2.1 flies. Each vertical bar represents a single sip. Flies were feeding with 10 mM sucrose mixed with 0.25% agarose (a) or 0.4% agarose (b). n = 16 for each group. (c, d) FlyPAD assay of sd-L-SS (vGluT-AD; iav-DBD) flies. Each vertical bar represents a single sip. Flies were feeding with 10 mM sucrose mixed with 0.25% agarose (c) or 0.4% agarose (d). n = 16 for each group. (e, f) FlyPAD assay of UAS-kir2.1flies. Each vertical bar represents a single sip. Flies were feeding with 10mM sucrose mixed with 0.25% agarose (e) or 0.4% agarose (f). n = 16 for each group. (TIF) [file pgen.1010562.s004.tif]

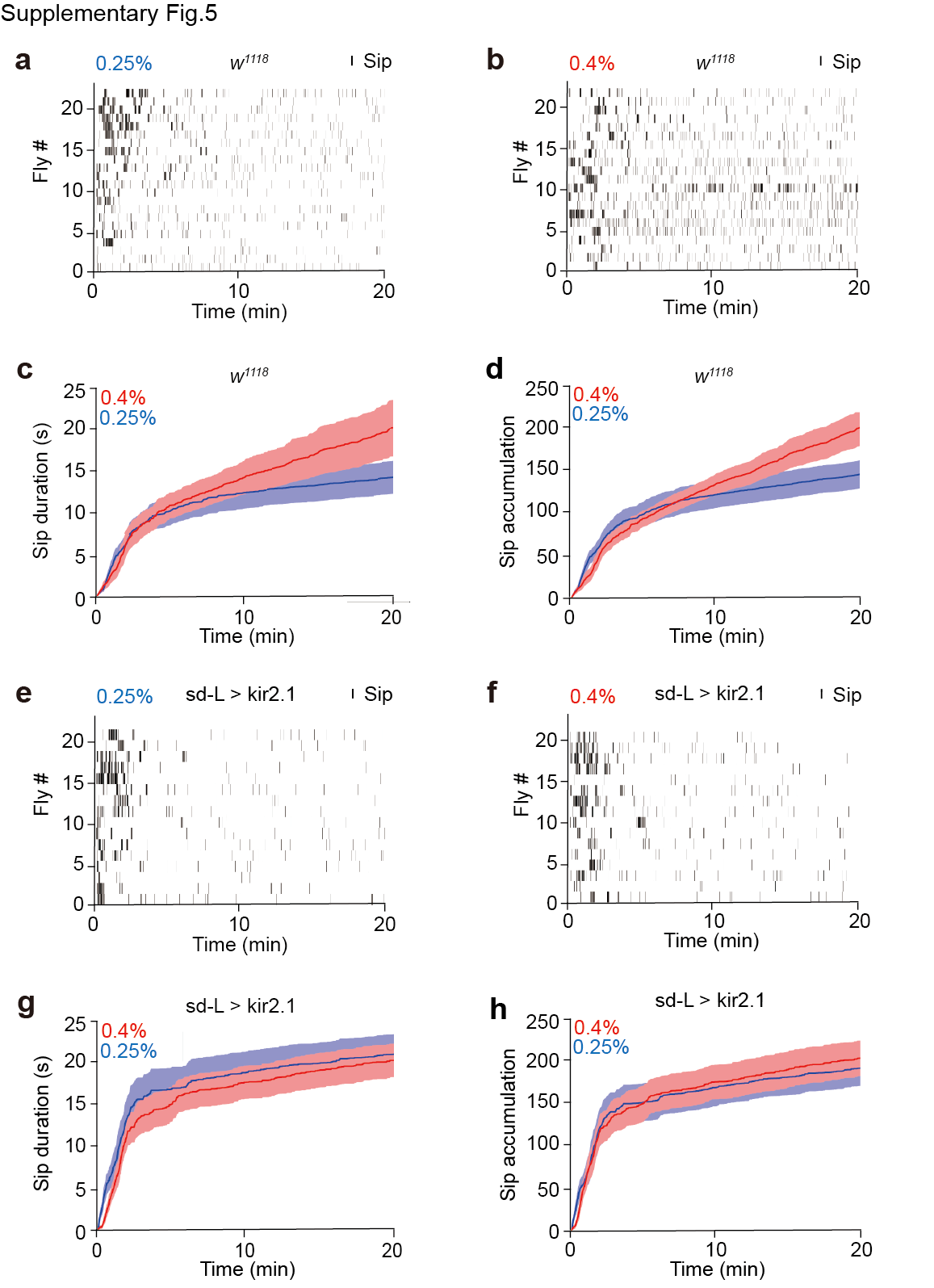

Supplement: S5 Fig — (a-d) FlyPAD assay of w1118 male flies. Each vertical bar represents a single sip (a, b). Flies were fed with plain agarose of 0.25% (a) and 0.4% (b). Cumulative sip durations on 0.25% or 0.4% agarose (c). Cumulative sip numbers on 0.25% or 0.4% agarose (d). n = 22 for each group. (e-h) FlyPAD assay of sd-L-SS > UAS-kir2.1 male flies. Each vertical bar represents a single sip (e, f). Flies were fed with plain agarose of 0.25% agarose (e) and 0.4% agarose (f). Cumulative sip durations on 0.25% or 0.4% agarose (g). Cumulative sip numbers on 0.25% or 0.4% agarose (h). n = 16 for each group. (TIF) [file pgen.1010562.s005.tif]

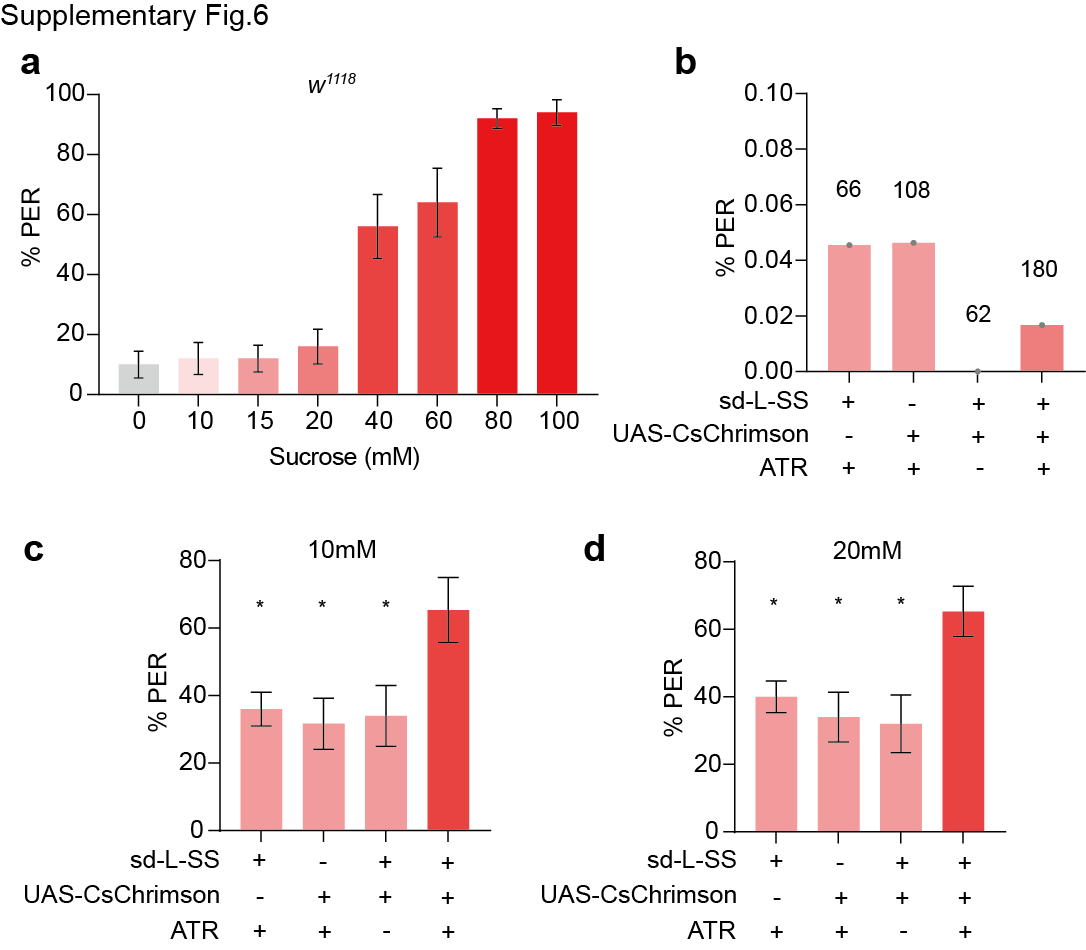

Supplement: S6 Fig — (a) PER assay of w1118 male flies with the increase of sucrose concentration. 8 different sucrose concentrations (0 ~ 100 mM) were used to induce PER response. Data are represented as mean ± SEM; n = 10 for each group. (b) PER response to water during optogenetic activation of sd-L neurons. We counted all the flies’ water response before and after the sugar test, n = 66, 108, 62 and 180 for each group. (c-d) PER assay of flies when sd-L neurons were activated using CsChrimson by exposure to 1 mW/cm2 light (595nm). All the legs were removed and tested with 10 mM sucrose (c) or 20 mM sucrose (d). Statistical test: one-way ANOVA with Dunnett’s correction for multiple comparisons against the sd-L-SS > UAS-CsChrimson with ATR group; mean ± SEM; n = 10 ~ 15 for each group. (TIF) [file pgen.1010562.s006.tif]

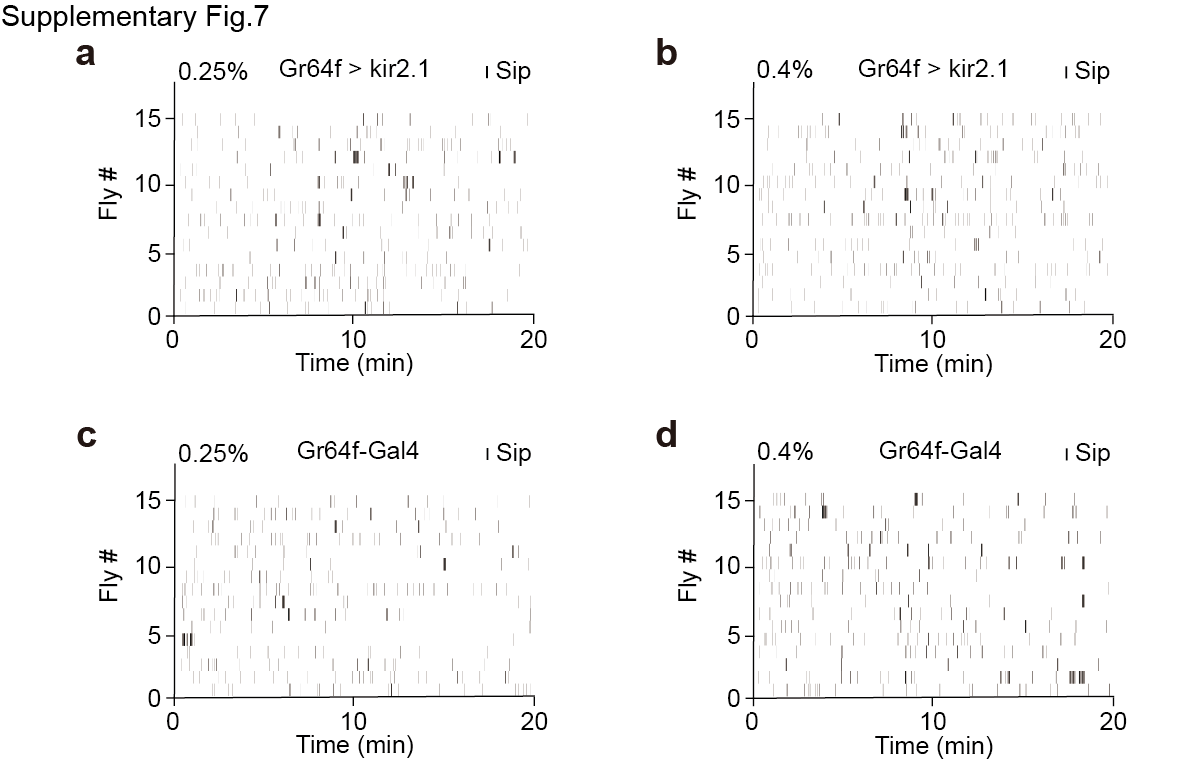

Supplement: S7 Fig — (a, b) FlyPAD assay of Gr64f-Gal4 > UAS-kir2.1 flies. Each vertical bar represents a single sip. Flies were fed with 10 mM sucrose mixed with (a) 0.25% agarose or (b) 0.4% agarose. n = 16 for each group. (c, d) FlyPAD assay of Gr64f-Gal4 flies. Each vertical bar represents a single sip. Flies were fed with 10 mM sucrose mixed with (c) 0.25% agarose or (d) 0.4% agarose. n = 16 for each group. (TIF) [file pgen.1010562.s007.tif]

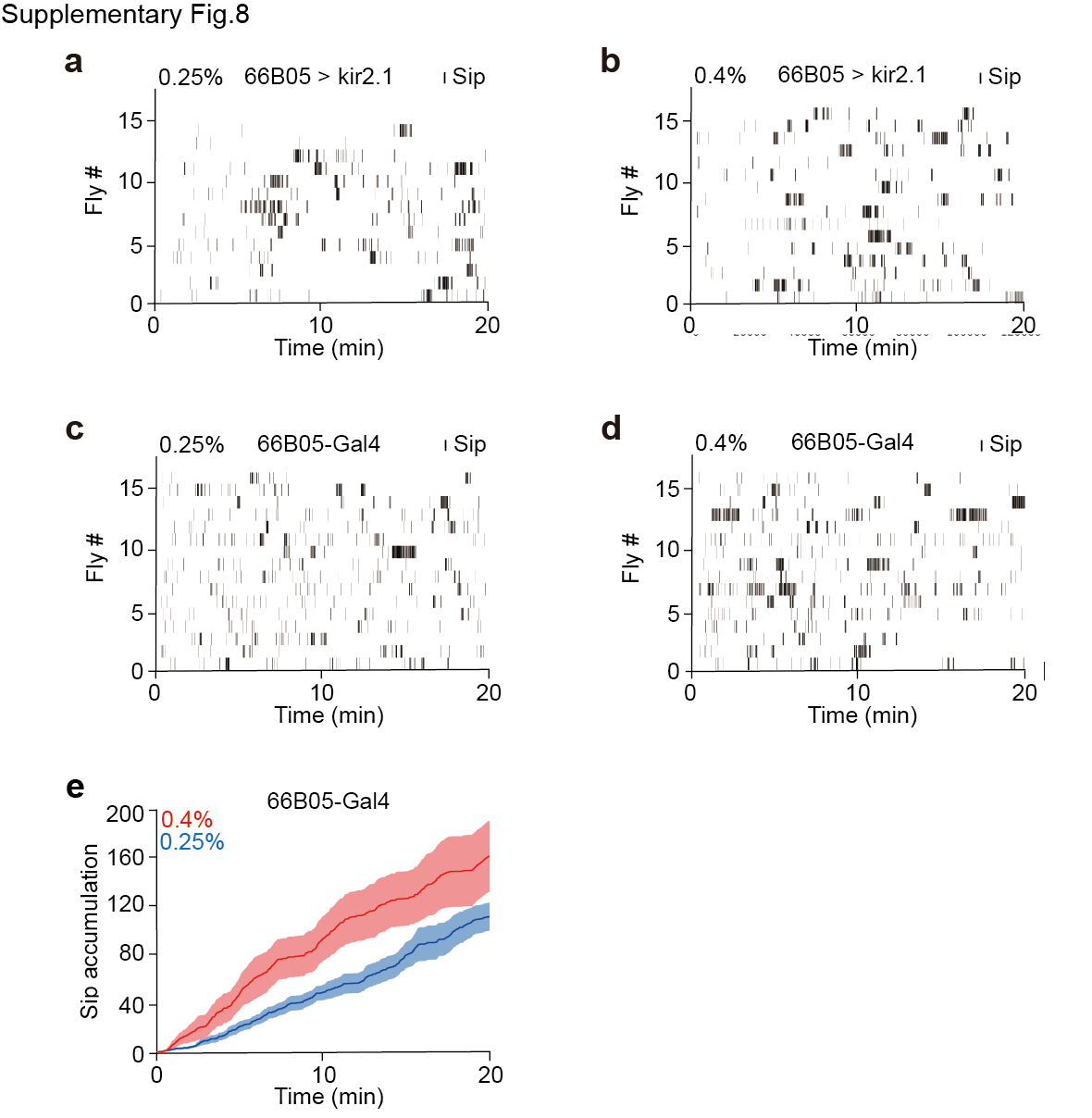

Supplement: S8 Fig — (a, b) FlyPAD assay of R66B05-Gal4 > UAS-kir2.1 flies. Each vertical bar represents a single sip. Flies were fed with 10 mM sucrose mixed with (a) 0.25% agarose or (b) 0.4% agarose. n = 14~16 for each group. (c, d) FlyPAD assay of R66B05-Gal4 flies. Each vertical bar represents a single sip. Flies were fed with 10 mM sucrose mixed with (c) 0.25% agarose or (d) 0.4% agarose. n = 16 for each group. (e) Cumulative sip numbers of R66B05-Gal4 flies in FlyPAD assay. Both 0.25% and 0.4% agarose containing 10mM sucrose, n = 16 for each group. (TIF) [file pgen.1010562.s008.tif]

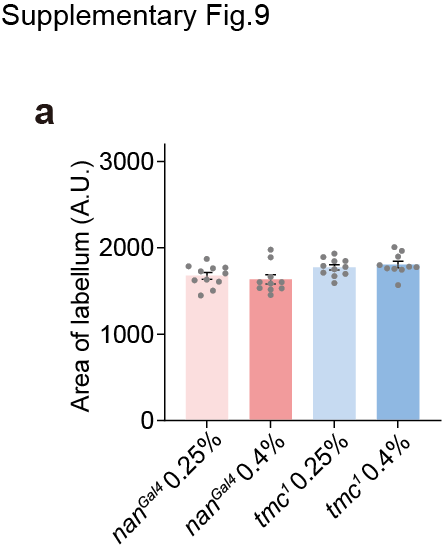

Supplement: S9 Fig — (a) Quantification of labellum spreading area of nangal4 and tmc1 when fed with 0.25% or 0.4% agarose containing 100 mM sucrose. Data are represented as mean ± SEM; n = 10 ~ 11 for each group. (TIF) [file pgen.1010562.s009.tif]
